# Rough surface Au@Ag core–shell nanoparticles to fabricating high sensitivity SERS immunochromatographic sensors

**DOI:** 10.1186/s12951-015-0142-0

**Published:** 2015-11-14

**Authors:** Qiangqiang Fu, Hongwu Liu Liu, Ze Wu, An Liu, Cuize Yao, Xiuqing Li, Wei Xiao, Shiting Yu, Zhi Luo, Yong Tang

**Affiliations:** Guangdong Province Key Laboratory of Molecular Immunology and Antibody Engineering, Department of Bioengineering, Jinan University, Guangzhou, 510632 People’s Republic of China; Integrated Optics and Biophotonics Laboratory, Department of Electronic Engineering, Jinan University, Guangzhou, 510632 People’s Republic of China; Institute of Biotranslational Medicine, Jinan University, Guangzhou, 510632 People’s Republic of China

**Keywords:** Rough surface core–shell Au@Ag nanoparticles, SERS, Immunochromatographic sensors, Haemoglobin, Cadmium ion

## Abstract

Immunochromatographic sensors (ICSs) are inexpensive, simple, portable, and robust, thus making ICSs commonplace in clinical diagnoses, food testing, and environmental monitoring. However, commonly used gold nanoparticles (AuNPs) ICSs have low sensitivity. Therefore, we developed highly sensitive surface enhanced Raman scattering (SERS) ICSs. To enhance the sensitivity of SERS ICSs, rough surface core–shell Au@Ag nanoparticles (RSAu@AgNPs) were prepared by coating silver on the surface of gold nanoflowers (AuNFs). Then these nanoparticles were used as SERS substrate in the SERS ICSs, after which the SERS ICSs were implemented to detect haemoglobin and heavy metal cadmium ion (Cd^2+^). The limit of detection (LOD) of the SERS ICSs for detecting haemoglobin was 8 ng/mL, and the linear range of the SERS ICSs was from 31.3 to 2000 ng/mL. The LOD of the SERS ICSs for detecting Cd^2+^ was 0.05 ng/mL and the linear analysis range was from 0.05 to 25 ng/mL. The cross reactivity of the SERS ICSs was studied and results showed that the SERS ICSs exhibited highly specific for detection of haemoglobin and Cd^2+^, respectively. The SERS ICSs were then used to detect haemoglobin (spiked in serum and in stool) and Cd^2+^ (spiked in tap water, river water, and soil leaching water), and the results showed high recovery. These characteristics indicated that SERS ICSs were ideal tools for clinical diagnosis and environmental pollution monitoring.

**Electronic supplementary material:**

The online version of this article (doi:10.1186/s12951-015-0142-0) contains supplementary material, which is available to authorized users.

## Background

Due to their low cost, robustness, convenience and rapidity, immunochromatographic sensors (ICSs) have been extensively used in medicine, agriculture, and over-the-counter personal use, such as pregnancy tests [1–5]. Many kinds of materials, such as colloidal gold [6], colloidal carbon [7], liposome [8, 9], quantum dots [10, 11], fluorescent nanoparticles [12], and organic fluorescent dyes [13] were used as racers in ICSs. Among the aforementioned materials, AuNPs were widely adopted, due to their vivid color, ease of synthesis, low cost, and excellent chemical stability. However, when compared with laboratory based methods, the sensitivity of most AuNPs ICSs to detect biomarkers was significantly less than other methods, such as enzyme-linked immunoassay (ELISA). Consequently, AuNPs ICSs are not particularly useful for detecting lower concentration biomarkers. Compared with AuNPs ICSs, fluorescent ICSs exhibit higher sensitivity (10-30 times higher than AuNPs ICSs). Currently used fluorescent tracers in ICSs are organic fluorescent dyes, fluorescent nanoparticles, and quantum dots. However, organic fluorescent dyes such as FITC are known to be photo-unstable and have relatively low fluorescence intensities. Even though fluorescent nanoparticles have high fluorescence intensity, excellent photo-stability, and high conjugation efficiency, both organic fluorescent dyes and fluorescent nanoparticles have a relatively small Stokes shift that often leads to fluorescent ICSs suffering from background interference. Another fluorescent tracer currently used in ICSs is quantum dots. This is because of the excellent features of quantum dots: they have very high levels of brightness, size-tunable fluorescence emission, narrow spectral line widths, large absorption coefficients, and excellent stability against photo-bleaching. However, the price of quantum dots is currently relatively high.

Surface-enhanced Raman scattering (SERS) based sensors provide the potential for rapid, high-throughput, sensitive detection. SERS have been used for cell imaging [14–16], tumor diagnosis [17–19], enzyme activity analysis [20, 21], nucleic acids analysis [22–24], gene mutations analysis [25], immune sensors [26, 27], and aptamer sensors [28, 29]. Over the past decade, the SERS field has witnessed many achievements, including a theoretical study of SERS [30, 31], the development of portable and high-performance Raman spectrometers [32–35], and the fabrication of highly sensitive, uniform, and reproducible SERS substrates [36, 37]. These important studies provide the basis for many SERS applications. AuNPs and silver nanoparticles (AgNPs) are the most widely used substrates for SERS. The SERS enhancement factor of AgNPs was demonstrated to be higher than that of AuNPs. It has been theoretically predicted and experimentally confirmed that sharp metallic protrusions and nano-gaps, called ‘hot spots’, are essential for a stronger SERS response [38–40]; these include: nanoflowers, nanosatellites, nanourchins, nanostars, etc. In addition, Au@Ag core–shell nanoparticles exhibit a higher SERS efficiency than AuNPS or AgNPs under near-infrared excitation [41]. Xie reported flower-like AuNPs (three-dimensional branched nanoparticles with more than 10 tips) that exhibited strong Raman enhancement factors [42]. During our research, to obtain higher enhanced efficient SERS substrate, we coated silver on AuNFs to prepare higher SERS efficiency rough surface Au@Ag core–shell nanoparticles (RSAu@AgNPs). These nanoparticles were used to fabricate the highly sensitive SERS ICSs. We used the developed SERS ICSs to detect haemoglobin and Cd^2+^. The results showed that SERS ICSs have a high sensitivity and high recovery. These characteristics indicated that SERS ICSs were ideal tools for clinical diagnosis and environmental pollution monitoring.

## Experimental

### Reagents and materials

Chloroauric acid (HAuCl_4_, 99.99 %), silver nitrate (99.80 %), HEPES, 4-mercapto-benzoic acid (4-MBA), and ascorbic acid were obtained from Sigma-Aldrich (Shanghai, China). Other metal powders were purchased from Merck Chemical (Darmstadt, Germany). Nitrocellulose (NC) membranes were purchased from Millipore (Shanghai, China). Plastic backing, conjugation pads, sample pads and absorbent pads were purchased from Shanghai JieNing Bio-tech (Shanghai, China). Anti-Cd^2+^-EDTA monoclonal antibodies (mAb) and BSA-Cd^2+^-EDTA were prepared in our laboratory (Additional file 1). A couple of Anti-haemoglobin monoclonal antibodies and haemoglobin were gifted from Guangzhou Weimi Bio-Tech CO., LTD.

### Equipment

A field-emission transmission electron microscope (TEM, Philips, Holland), centrifuge (Beckman, Germany), and ICP-MASS (Thermo-Fisher, USA) were used. An XYZ 3200 series dispense system (Bio-Dot Scientific Equipment, Pvt. Ltd.), a programmable HGS201 strip cutter (purchased locally in Shanghai, China), and an Advantage 785 Near Infrared (NIR) Raman Spectrometer (SciAps, Inc.) were also used in this study (laser power60 mW).

### Preparation of RSAu@AgNPs

AuNFs were synthesized following a previous research paper [42]. In a typical experiment, 0.2 mL of 25 mM HAuCl_4_ was added to 10 mL of a 20 mM HEPES solution (pH 7.4). The formation of AuNFs was indicated when the initially light-yellow mixture changed to purple within approximately 30 min. The AuNFs were then stored at 4 °C until further use. RSAu@AgNPs were synthesized as follows: 40 µL of 0.1 M NaOH and 30 µL of 0.1 M ascorbic acid were added to 1 mL of the AuNFs. After shaking vigorously, 400 μL of 10 mM AgNO_3_ was added to the above solution. Following this addition, the mixture was again shaken vigorously and the color of the solution changed rapidly from purple to yellow, indicating the formation of RSAu@AgNPs. Preparations of AuNPS, AgNPs, and Au@AgNPs are displayed in Additional file 1.

### Preparation of mAb-RSAu@AgNPs-4MBA

A total of 0.2 μL of 10 mM 4-MBA contained in ethanol was added to 1 mL of the prepared RSAu@AgNPs, which was then shaken vigorously for 1 h. Then, 3 μL of 1 mg/mL monoclonal antibody (mAb) and 15 μL of 1 mg/mL PVP were added simultaneously to the above-mentioned solution and were shaken vigorously. After incubating at room temperature for 30 min, 100 μL of 10 mg/mL PVP was used to cover the surface of the RSAu@AgNPs for 1 h. The resulting colloid was centrifuged at 7000 rpm for 10 min. Then, this supernatant was discarded and the remaining pellet was suspended in 200 μL of dilution buffer [15 mM PB buffer (pH 8.0) containing 1 % (w/v) BSA, 20 % (w/v) sucrose, 20 % (w/v) trehalose, 1 % (w/v) Tween-20, and 0.02 % sodium azide]; it was then stored at 4 °C for further use.

### Fabrication of SERS ICSs

Prepared SERS ICSs for detecting Cd^2+^: coating antigen Cd^2+^-EDTA-BSA (0.1 mg/mL) was dispensed on the specified area of an NC membrane that was designated as the test line (T-line) by using an automatic dispenser with a volume of 1 μL/cm. The NC membrane was dried at 37 °C for 24 h. Sample pad was pre-treated with 0.01 M PBS buffer (pH 7.2), containing 0.5 % (w/v) BSA and 2 % Triton X-100. Using a dilution buffer, the mAb-RSAu@AgNPs-4MBA was diluted 64 times and then dispensed on the conjugate pad using the automatic dispenser with a volume of 2 μL/cm. After the pads were dried at 37 °C for 2 h, all components of the SERS ICSs were assembled with 2 mm overlaps. These stacks were then cut into ICSs strips and placed into plastic housings. Prepared SERS ICSs for detecting haemoglobin: mAb-RSAu@AgNPs-4MBA was diluted 32 times and then dispensed on NC membrane at T-line. Other similar processes to SERS ICSs for detecting Cd^2+^ were applied.

### Performance of the SERS ICSs

For detecting haemoglobin: A series of concentrations of a haemoglobin solution (60 μL) in PBS was detected using the SERS ICSs. After 15 min, Raman signals of these ICSs were recorded using a portable Raman spectrometer with an integration time of 20 s. For detecting Cd^2+^: Cd^2+^ solution was diluted to a series of concentrations by 50 nM EDTA-Na_2_ and detected by using SERS ICSs. After 15 min, the Raman signals of these ICSs were recorded as mentioned above. SERS ICSs detection of haemoglobin or Cd^2+^ in spiked samples is displayed in Additional file 1. Additionally, experimental procedures that study the specificity and recovery of SERS ICSs are displayed in Additional file 1.

## Results and discussion

### Synthesis and characterization of nanoparticles

**Fig. 1:**
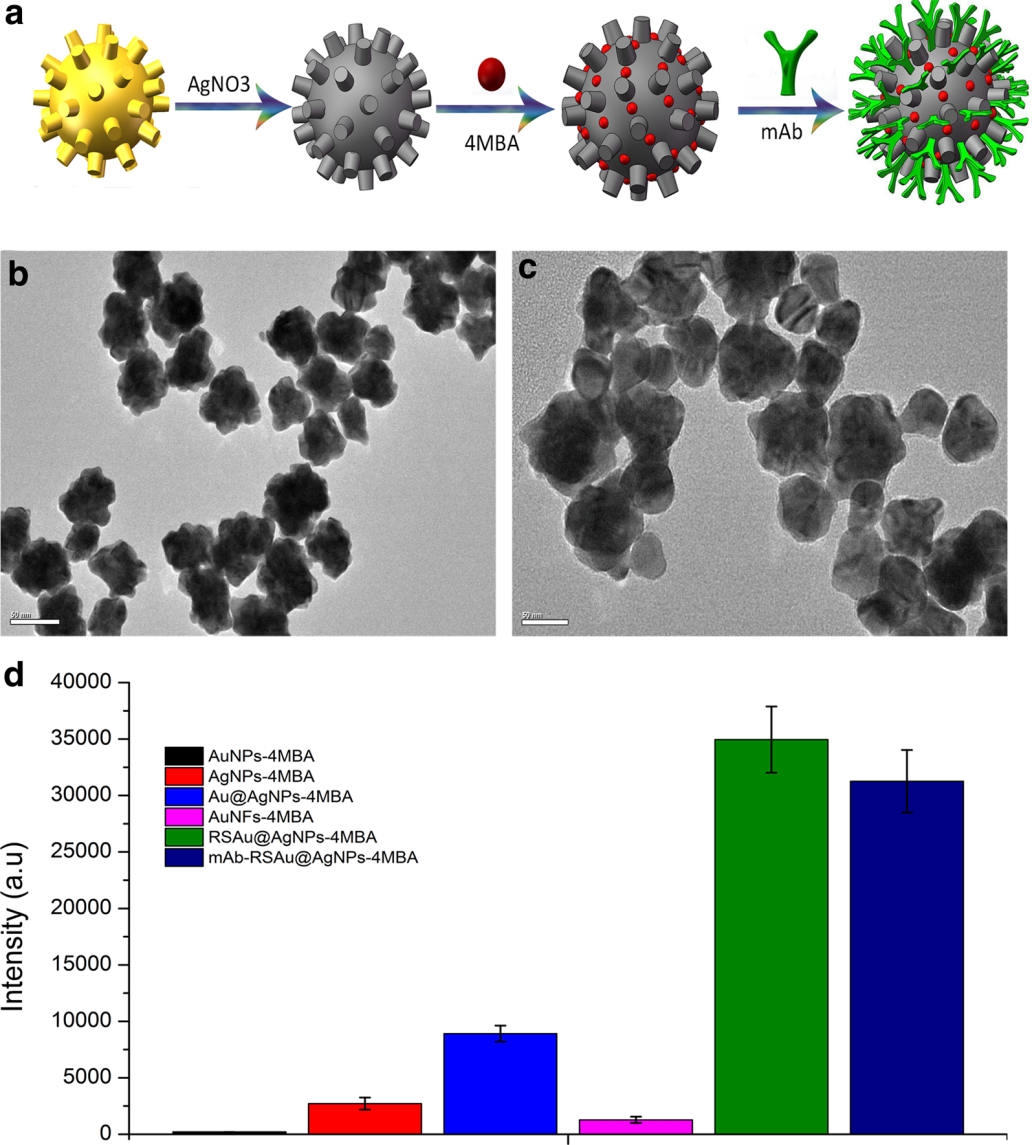
Synthesis and characterization of mAb-RSAu@AgNPs-4MBA. **a** Schematic diagram of synthesized mAb-RSAu@AgNPs-4MBA. **b**–**c** TEM images of AuNFs and RSAu@AgNPs, *scale bars* were 50 nm. **d** Comparisons: SERS efficiency of AuNFs-4MBA, RSAu@AgNPs-4MBA and mAb-RSAu@AgNPs-4MBA, AuNPs-4MBA, AgNPs-4MBA and Au@AgNPs-4MBA. The Raman signal was detected in 96-well micro-plates, and the exposure time was 20 s

AuNFs were synthesized using HEPES to reduce the chloroauric acid and were coated with a silver shell by reducing AgNO_3_ to elemental silver. We optimized the SERS efficiency of RSAu@AgNPs and showed that RSAu@AgNPs exhibited higher SERS efficiency by adding 400 μL 10 mM AgNO_3_ in 1 mL AuNFs (Additional file 1: Figure S4). Figure 1b–c show typical TEM images of the AuNFs, RSAu@AgNPs. Compared with AuNPs (Additional file 1: Figure S1), AgNPs (Additional file 1: Figure S2) and Au@AgNPs (Additional file 1: Figure S3), the RSAu@AgNPs surfaces are much rougher (Fig. 1b). AuNFs exhibited an SPR peak at 560 nm; whereas, the RSAu@AgNPs and mAb-RSAu@AgNPs-4MBA presented an SPR peak at 420 nm, indicating that the silver had successfully coated the surfaces of AuNFs (Additional file 1: Figure S6). Figure 1d shows the Raman signal intensity of the reporter molecule 4-MBA enhanced by the AuNFs, AuNPs, Au@AgNPs, RSAu@AgNPs and mAb-RSAu@AgNPs-4MBA. To accurately assess the SERS activity of these nanomaterials, the same amount of 4-MBA was added to an equal volume of each of the nano-materials with the same concentration. After optimization of 4-MBA concentration conjugate with RSAu@AgNPs-4MBA (Additional file 1: Figure S5), 0.2 μL of 10 mM 4-MBA was mixed with 1 mL of nanomaterials at room temperature. After 1 h, the SERS spectra were detected with exposure times of 20 s. The SERS spectrum of 4-MBA was characterized by peaks at 1077 cm^−1^. The SERS intensities of the RSAu@AgNPs-4MBA and mAb-RSAu@AgNPs-4MBA were considerably higher than those of the AuNPs-4MBA, AuNFs-4MBA, AgNPS-4MBA, and Au@AgNPs-4MBA. These results indicated that core–shell structures and rough surfaces give higher SERS efficiency to RSAu@AgNPs. These results are consistent with previous reports [41, 43].

### The SERS ICSs to detect haemoglobin

**Fig. 2:**
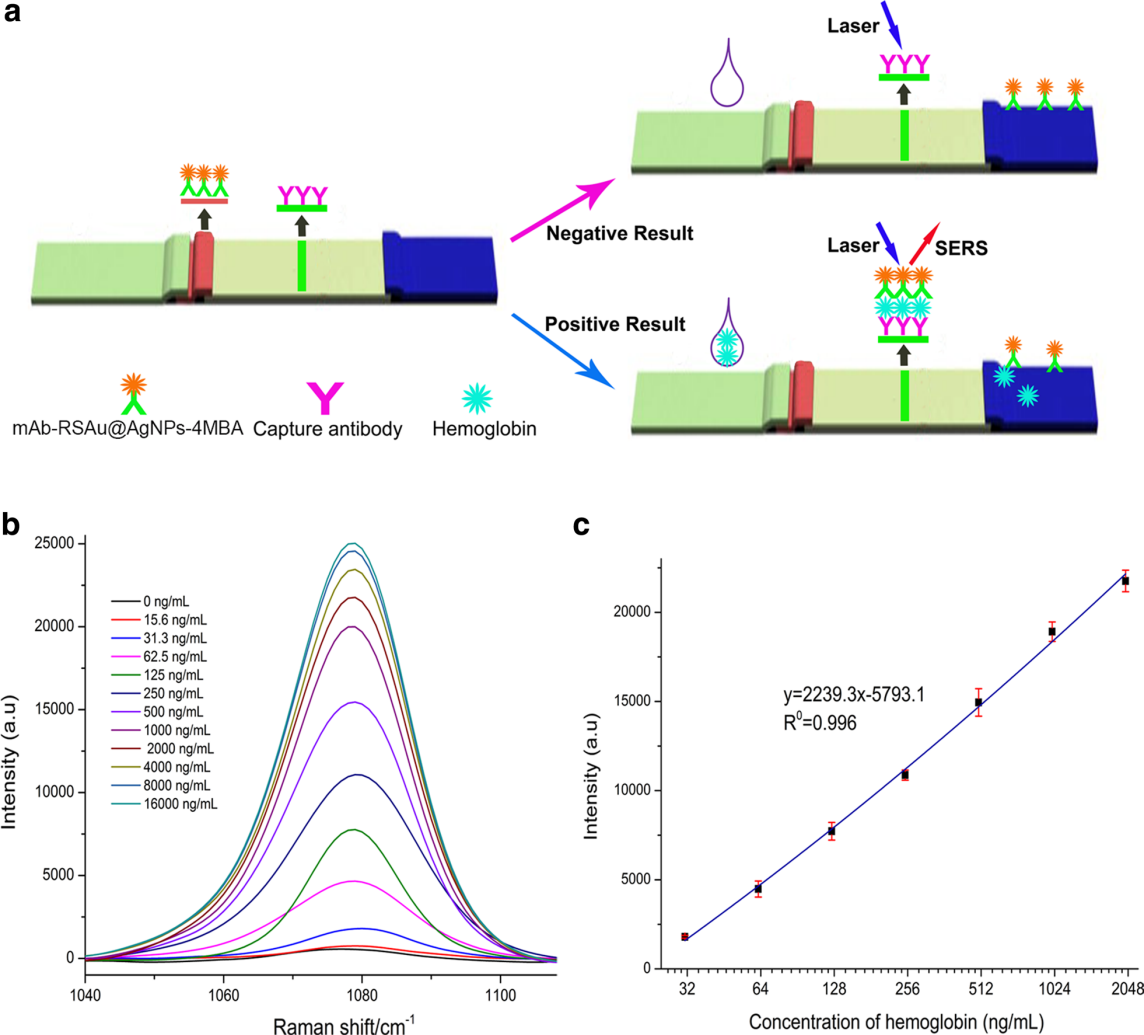
**a** Schematic diagram of the SERS ICSs for detecting haemoglobin. The SERS ICSs consists of five overlapping layers: absorption pad, NC membrane, conjugation pad and sample pad, which were placed on a plastic backing. Capture mAb was dispensed at T-line. When the SERS ICSs detected negative samples, Raman signal at T-line was weak; whereas, when the SERS ICSs detected positive samples, Raman signal at T-line was strong. **b** Concentration dependent SERS spectra of SERS ICSs obtained from detecting different concentrations of haemoglobin: The entire SERS spectra are shown in Additional file 1: Figure S10–21. Detailed vibrational assignments of Raman peaks are presented in Additional file 1: Table S1. **c** Calibration curve of SERS ICSs for the detection of haemoglobin

In clinical studies, hemoglobin is an important biomarker for diagnosing intestinal bleeding. In this study, we prepared SERS ICSs and used them to detect haemoglobin. The SERS ICSs for detecting haemoglobin consisted of five components (from top to bottom): (a) a sample pad for applying samples, (b) a conjugate pad for loading mAb-RSAu@AgNPs-4MBA, (c) a 25 mm NC membrane acting as the chromatography matrix, (d) an absorbent pad serving as the liquid sink, and (e) a plastic backing for supporting all the components (Fig. 2a). The capture mAb was dispensed on the NC membrane at T-line. The principle of the SERS ICSs is shown in Fig. 2b–c. When negative samples (not containing analytes) were applied, the liquid samples dispersed mAb-RSAu@AgNPs-4MBA that were preloaded on the conjugation pad and made the mAb-RSAu@AgNPs-4MBA migrate toward the absorbent pad. Hemoglobin did not bind with mAb-RSAu@AgNPs-4MBA; therefore, when samples reached T-line zone, mAb-Au@AgNPs-4MBA could not bind to the coating mAb at T-line. Subsequently, a weak SERS signal at T-line was detected. In contrast, when a certain amount of haemoglobin solution was applied to the sample pad, haemoglobin would first bind to the mAb-RSAu@AgNPs-4MBA; these nanoparticles were then captured by mAb at T-line and a strong Raman signal was detected. Raman signal intensity of the ICSs at T-line increased, as concentrations of haemoglobin elevated. To facilitate the analysis of the detection results, we chose Raman intensity at the peak of 1077 cm^−1^ as the test signal and the integration times of the ICSs test were maintained at 20 s.

The surfactant triton X-100 accelerated the diffusion speed of mAb-RSAu@AgNPs-4MBA at NC membrane, thereby, reducing the time taken for SERS to detect haemoglobin. However, a high concentration of surfactant triton X-100 reduced the amount of time that mAb-RSAu@AgNPs-4MBA stayed at T-line and decreased the sensitivity of SERS ICSs. Considering the detection time and sensitivity of the SERS ICSs, 2 % triton X-100 was contained in sample pad treatment agent (Additional file 1: Figure S7). Following these procedures, concentrations of mAb-RSAu@AgNPs-4MBA that dispersed on conjugation pad, which impacted the performance of ICSs, were optimized. A high concentration of mAb-RSAu@AgNPs-4MBA dispersed on conjugation pad enhanced the sensitivity of SERS ICS; however, this also may have increased the background SERS signal on nitrocellulose membrane. Considering the background SERS signal and sensitivity of the SERS ICSs, mAb-RSAu@AgNPs-4MBA was diluted 32 times and then dispersed on conjugation pad (Additional file 1: Figure S8).

The results for detecting a series of concentrations of haemoglobin are shown in Fig. 2b–c. The detection time was chosen as 15 min (Additional file 1: Figure S9). When negative samples were applied to the SERS ICSs, the Raman signal intensity at T-line was 492 a.u. When haemoglobin concentrations were higher than 15.6 ng/mL, SERS signal at T-line showed a gradual increase. When haemoglobin concentrations were higher than 16,000 ng/mL, SERS signal at T-line remained at approximately 24,500 a.u. The calibration curve of SERS ICSs for detecting haemoglobin had a positive slope, with a linear detection range from 32.3 to 2000 ng/mL. The LOD of SERS ICSs was 8 ng/mL, calculated by a concentration response to the SERS intensity (SRES intensity of negative sample + 3 × SD).

### The SERS ICSs for detecting Cd^2+^

**Fig. 3:**
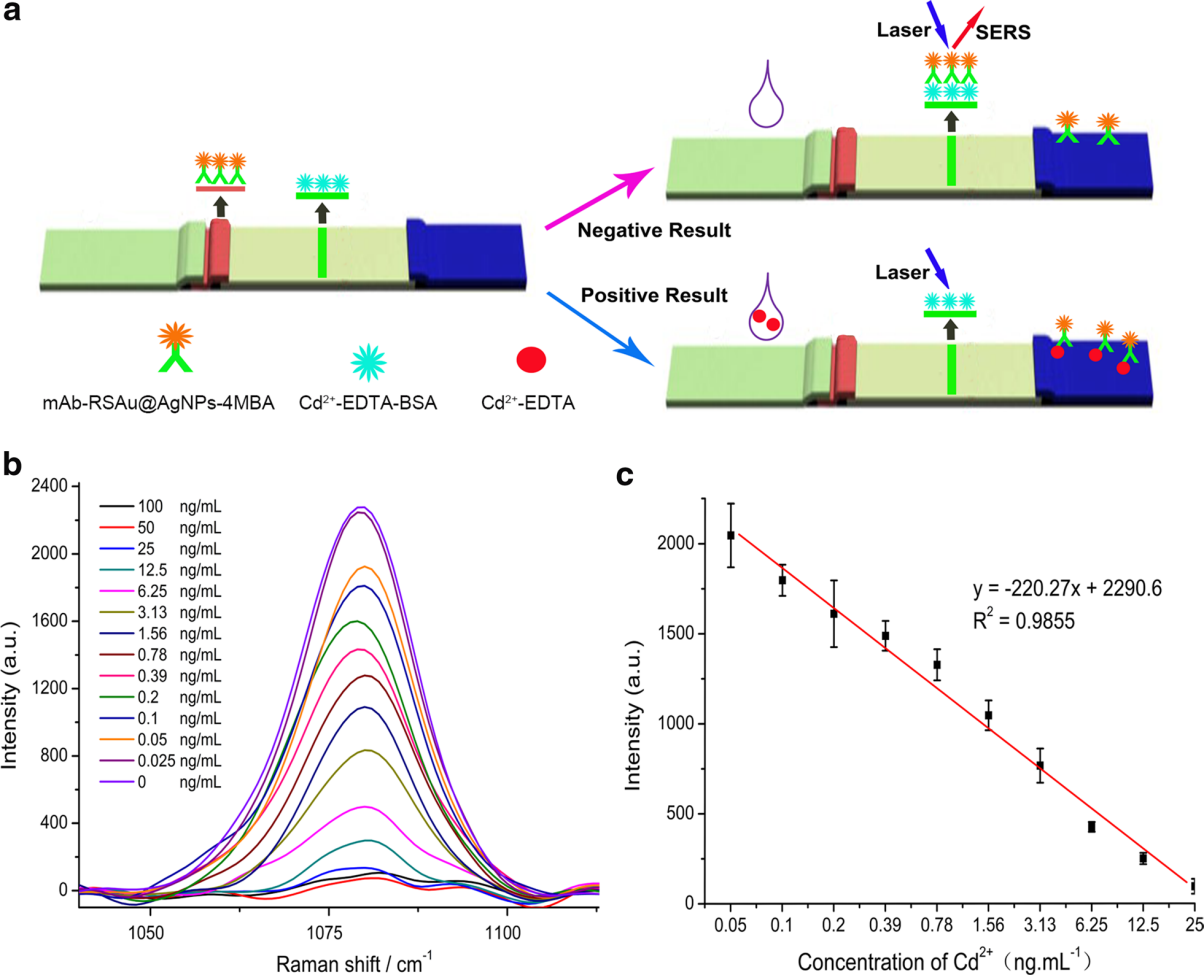
**a** Schematic diagram of the SERS ICSs for detecting Cd^2+^. **b** Concentration dependent SERS spectra of the ICSs obtained from the precipitates that corresponded to different concentrations of Cd^2+^. The entire SERS spectra are shown in Additional file 1: Figure S24–38. **c** Calibration curve of SERS ICSs for the detection of Cd^2+^

To demonstrate that SERS ICSs were appropriate for detecting small molecules, we further used them to detect heavy metal cadmium (Cd^2+^). Cd^2+^ is believed to have a biological half-life of greater than 10 years in the human body. Humans are exposed to cadmium predominantly through the ingestion of cadmium contaminated food, water, and soil, or through the inhalation of cadmium-containing dusts. After ingestion, cadmium accumulates in the kidneys, liver, lungs, and gastrointestinal tract, where it can then cause progressively toxic effects, including cancer and renal damage [44, 45]. Because Cd^2+^ is too small to be recognized by antibodies, EDTA-2Na was selected to chelate Cd^2+^ to form a specific hapten. To prepare the complete antigen, iEDTA was used to conjugate Cd^2+^ and link the carrier proteins. To prepare SERS ICSs for detecting Cd^2+^: Cd^2+^-EDTA-BSA was dispersed at T-line and anti-Cd^2+^-EDTA mAb labeled RSAu@AgNPs-4MBA were dispersed on conjunction pad. The principle of the SERS ICSs that detected Cd^2+^ is shown in Fig. 3. When negative samples (containing no analytes) were applied, mAb-RSAu@AgNPs-4MBA were dispersed in samples and migrated toward the absorbent pad. When samples reached T-line zone, mAb-RSAu@AgNPs-4MBA bound to Cd^2+^-EDTA-BSA at T-line and the SERS signal was detected. In contrast, when a certain amount of positive sample solution was applied, Cd^2+^-EDTA would first bind to the mAb-RSAu@AgNPs-4MBA. Then, the amount of mAb-RSAu@AgNPs-4MBA that bound at T-line decreased and the intensity of the Raman signal at T-line became weaker. The Raman signal intensity at T-line decreased, as the concentration of Cd^2+^ in the samples increased. Exposure times of the Raman spectrometer were maintained at 20 s. The results for detecting different concentrations of Cd^2+^ are shown in Fig. 3b–c. When negative samples were applied to the ICSs, the Raman signal intensity at T-line reached 2300 a.u. The SERS signal intensity began decreasing when Cd^2+^ concentrations were greater than 0.05 ng/mL, and SERS signal intensity gradually decreased when Cd^2+^ concentrations increased. When Cd^2+^ concentrations were higher than 25 ng/mL, the signal at T-line remained at approximately 100 a.u. As shown in Fig. 3c, the calibration curve of SERS ICSs for detecting Cd^2+^ shows a negative slope and a linear detection range between 0.05 and 25 ng/mL. The LOD of SERS ICSs was 0.05 ng/mL, which was calculated by concentration response to the SERS intensity (SERS intensity of negative sample – 3 × SD). A thermal accelerated test was used to study the storage time of SERS ICSs for detecting Cd^2+^ ion. These SERS ICSs were stored at 37 °C and SERS intensity of the ICSs was kept constant for 30 days. According to Arrhenius equation, the developed SERS ICSs could be stored for 120 days at 25 °C (Additional file 1: Figure S43).

### Comparison of SERS ICSs and AuNPs ICSs for detecting haemoglobin and Cd^2+^

A comparison of SERS ICSs and AuNPs ICSs based on the same antigen and antibody is shown in Table 1. The sensitivity of SERS ICSs is higher than AuNPs ICSs and has a wider dynamic detection range.

**Table 1:** Comparison of SERS ICSs, AuNPs ICSs

Analytes	Methods	Detection range (ng/mL)	Limit of detection (ng/mL)	Detection time (min)
Haemoglobin	SERS ICSs	31.3–2000	8	15
	AuNPs ICSs	2000–16,000	1000	15
Cd^2+^	SERS ICSs	0.05–25	0.05	15
	AuNPs ICSs	25–400	25	15

Details of AuNPs ICSs are presented in the Additional file 1: Figure S34–38

### Specificity of the SERS ICSs

**Fig. 4:**
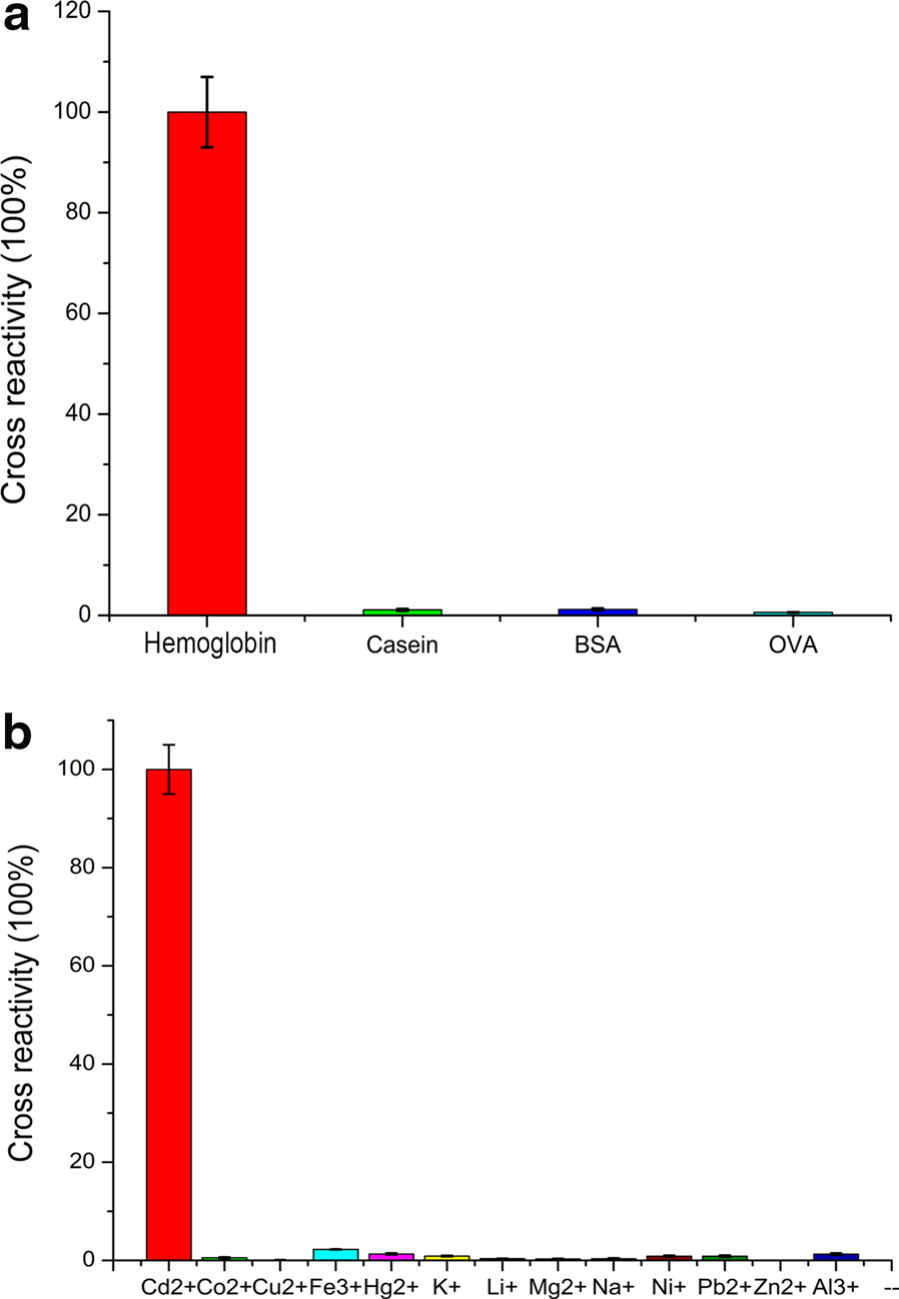
Specificity of the SERS ICSs. **a** Specificity of the SERS ICSs for detecting hemoglobin. **b** Specificity of the SERS ICSs for detecting Cd^2+^

The cross reactivity of SERS ICSs is shown in Fig. 4. For studying specificity of SERS ICSs that detect haemoglobin, 20,000 ng/mL of thrombin, casein, BSA, and OVA were dissolved in PBS and then detected using the SERS ICSs. The results indicated that the SERS ICSs had low cross reactivity with casein, BSA, and OVA. While researching the specificity of SERS ICSs used to detect Cd^2+^, various metal ions (25 ng/mL) were spiked in water samples and then detected by using SERS ICSs. The results indicated that the developed ICSs had cross-reaction rates of 0.55, 0.02, 2.24, 1.32, 0.89, 0.38, 0.30, 0.36, 0.85, 0.87, 0, and 1.28 % with Co^2+^, Cu^2+^, Fe^2+^, Hg^2+^, K^+^, Li^+^, Mg^2+^, Na^+^, Ni^+^, Pb^2+^, Zn^2+^, and Al^3+^ respectively. These results indicated that the developed SERS ICSs had high specificity.

### Recovery of SERS ICSs for detecting haemoglobin and Cd^2+^

While studying the recovery of SERS ICSs for detecting haemoglobin, SERS ICSs were also used to detect haemoglobin that had been spiked in blood and stool samples. Additionally, while researching the recovery of SERS ICSs for detecting Cd^2+^, SERS ICSs were used to detect Cd^2+^ that were spiked in tap water, river water, and soil leaching water. Table 2 summarizes the recovery of SERS ICSs. Recoveries of SERS ICSs that detected haemoglobin spiked in serum and stool varied from 5.06 to 14.8 %. For detecting Cd^2+^ spiked in tap water, Pearl River water, and soil leaching water, the recoveries of SERS ICSs were ranged from 90.67 to 110.0 %, 96.0 to 120.0 % and 92.00 to 116 %, respectively. These correlated results that haemoglobin was detected by using ELISA and Cd^2+^ was detected by using ICP-MASS.

**Table 2:** Recovery of SERS ICSs for detecting haemoglobin and Cd^2+^

Analytes	Samples	Spiked concentrations (ng/mL)	Detected by SERS ICSs (ng/mL)	Recovery of ICS (%)^b^	Coefficient of variation (%)^c^	Detect by ELISA/ICP-MASS (ng/mL)
Haemoglobin	Stool processed solution	1000	840 ± 125	84	14.8	920.3 ± 53.3
		800	751 ± 38	93.9	5.06	810 ± 61
		200	183 ± 14	91.5	7.65	188.6 ± 11.3
		50	47 ± 3.2	94	6.8	46.6 ± 3.9
	Serum	1000	1090 ± 60.4	109	5.54	990 ± 44.6
		500	518 ± 18.2	103.6	3.51	481.4 ± 38.4
		100	111 ± 9.1	111	8.2	99.5 ± 7.3
Cd^2+^	Pearl river water	250	246.7 ± 4.4	98.68	1.78	226.7 ± 40.4
		50	54.3 ± 8.1	108.67	14.99	54.3 ± 8.1
		10	11.0 ± 2.6	110.00	24.05	11.0 ± 2.6
		2	1.9 ± 0.2	92.50	11.78	1.9 ± 0.2
	Tap water	250	246.7 ± 10.0	96.00	4.17	246.7 ± 10.0
		50	55.0 ± 6.6	110.00	11.92	55.0 ± 6.6
		10	12.0 ± 1.7	120.00	14.43	12.0 ± 1.7
		2	2.1 ± 0.2	106.67	10.83	2.1 ± 0.2
	Soil leaching water	250	230.0 ± 26.5	92.00	11.50	230.0 ± 26.5
		50	58.0 ± 2	116.00	3.45	58.0 ± 2
		10	10.3 ± 2.5	103.33	24.35	10.3 ± 2.5
		2	2.1 ± 0.2	105.83	11.65	2.1 ± 0.2

Results are expressed as the mean ± SD (n = 3)

^a^SERS ICSs procedure is described in the text

^b^Recovery (%) = (detected concentration/spiked concentration) × 100

^c^Coefficient of variation (CV) calculated as CV = (SD/mean) × 100 %

## Conclusion

In conclusion, because of their core–shell structures and rough surfaces, RSAu@AgNPs have a high Raman enhancement efficiency, and be used to fabricate high sensitivity SERS ICSs for detecting haemoglobin and Cd^2+^. When compared with AuNPs ICSs, the developed SERS ICSs possessed a higher sensitivity, which indicated that SERS ICSs could detect lower concentrations of analytes when AuNPs ICSs were unavailable. In actual testing, pH, ionic strengths, and impurities in samples often affected the stability and accuracy of detection results. Therefore, by using SERS ICSs, samples could be diluted many times to reduce the effects of pH, ionic strength, and impurities. These results indicate that SERS ICSs are ideal tools for clinical diagnoses, food safety testing, and environmental pollution monitoring.

## Additional file

10.1186/s12951-015-0142-0 Protein purification.
