# Supplementary material for: Rough surface Au@Ag core–shell nanoparticles to fabricating high sensitivity SERS immunochromatographic sensors
Source: J Nanobiotechnology. 2015 Nov 14;13:81. doi: 10.1186/s12951-015-0142-0 (PMC4650504; doi:10.1186/s12951-015-0142-0)
Supplement: Supplementary file 1 — 10.1186/s12951-015-0142-0 Protein purification. [file 12951_2015_142_MOESM1_ESM.docx]

Supplementary Data

Rough Surface Au@Ag core-shell nanoparticles to fabricating high sensitivity SERS immunochromatographic sensors

Qiangqiang Fu^a,1^, Hongwu Liu^b,1^, Ze Wu^a^, An Liu, Caifeng Lan^a^,Cuize Yao^a^, Xiuqing Li^a^, Jiajie Liang^a^, Zhi Luo^b,∗^, Yong Tang^a,c,∗^

^a^Department of Bioengineering, Guangdong Province Key Laboratory of Molecular Immunology and Antibody Engineering, Jinan University,Guangzhou 510632, PR China.

^b^Department of Electronic Engineering,  Integrated Optics and Biophotonics Laboratory,

Jinan University, Guangzhou 510632, PR China

^c^ Institute of Biotranslational Medicine, Jinan University, Guangzhou, 510632, PR China.

^∗^Corresponding authors: Fax: (+86)-20-85227003; Tel: (+86)-20-85227003; E-mail: [tyjaq7926@163.com](mailto:tyjaq7926@163.com). Fax:86-20-85220415; Tel: 86-20-85220231; [zhluocn@gmail.com](mailto:zhluocn@gmail.com)

^1^These authors contributed equally to the manuscript

1. **Experimental**

### Preparation of the coating antigen

The low-molecular-weight Cd was too small to elicit an immune response. Therefore, iEDTA, a highly effective bifunctional chelating agent, was selected to conjugate Cr and carrier proteins. EDTA–protein complexes were prepared by an appropriate modification method. 0.4 mg of iEDTA was dissolved in 100 mL of dimethyl sulfoxide (DMSO), and 3.4 mg of BSA was dissolved in 1.0 mL Tris-HCl buffer (0.1 M, pH 8.5). The reaction mixtures were adjusted to pH 9.0 with triethylamine and stirred for 24 h at 25 °C. Small unconjugated molecules were removed by buffer exchange with centricon-30 micro-concentrators. The conjugates were washed with metal-free Tris–HCl buffer (0.1 M, pH 7.4), and then 80 μL of chromic chloride (1 M) was added dropwise with gentle stirring to the purified EDTA-protein conjugate while stirring gently. The complex reaction was allowed to proceed for 4 h at 25 °C. Finally, the unreacted Cd ions were removed as described.

**Hybridoma production and antibody purification**

Four 6-week-old female BALB/c mice were immunized with Cd^2+^-iEDTA-BSA on days 0, 14, and 28. Three cell lines secreting monoclonal antibodies against Cd^2+^-iEDTA were established by using the cell fusion technology, indirect ELISA and competitive ELISA. Hybridomas synthesizing and secreting metal-specific antibodies were subcloned twice by limiting dilution. Positive hybridoma clones were subcultured and frozen. The best cell line coded as 5G11C2 was selected to produce ascitic antibodies by injecting 106 hybridoma cells in mice. The mAb were precipitated with saturated ammonium sulfate and dissolved in phosphate buffer (0.002 M, pH 7.4) and then were dialyzed against the same buffer at 4 °C for 2 days. The IgG proteins were isolated by affinity chromatography with an immobilized protein G column. The antibody concentration was determined by using the BCA protein assay.

**Specificity of SERS ICSs**

To evaluate the specificity of the SERS ICSs for detect hemoglobin, BSA OVA and casein were chosen as controls each with concentrations of 2000 ng/mL. To evaluate the specificity of the SERS ICSs, a series of metal ions were chosen as controls each with concentration of 25 ng/mL.

**Recovery of SERS ICSs**

To evaluate the recovery of SERS ICSs for detect hemoglobin, a series concentrations of hemoglobin were spiked in 1g stool and serum, and then add 1 mL PBS. After 15 min, samples were centrifuged and the suspension was analysis by SERS ICSs. To evaluate the recovery of SERS ICSs for detect Cd^2+^, A series of metal ions solutions that contain with 50 nM EDTA-Na2 were analysis by the SERS ICSs as mentioned above.

### Preparation of AuNPs

AuNPs with a mean particle diameter of 20 nm were produced by reduction of gold chloride with 1% sodium citrate. Briefly, 2 mL of 1% gold chloride trihydrate solution in superpurified water was heated to boiling, and then 4.0 mL of 1% sodium citrate solution was added quickly into the solution under stirring over a period of 20 min. Gradually, the color changed from light yellow to brilliant red. After the color change, the solution was boiled for another 20 min to complete the reduction of gold chloride, cooled and stored at room temperature.

### Conjugation and purification of mAb-AuNPs

The pH of AuNPs (10 mL) was adjusted to 8.5 with 0.25 M K_2_CO_3_ and then added 10 μL of 1 mg/mL mAb. After 30 min, 1 mL of 10% BSA was added to block the excess site of AuNPs. incubated for 30 min, and finally centrifuged at 10,000 rpm for 15 min to remove the unconjugated antibodies. After removal of the supernatant, precipitate was suspended with 1 dilution buffer [15 mM PB buffer (pH8.0) containing 1% (w/v) BSA, 20% (w/v) sucrose, 20% (w/v) trehalose, 1% (w/v) Tween-20, and 0.02% sodiumazide], and stored at 4 °C until use.

**Fabrication of AuNPs** **ICSs.**

For detect Cd^2+^: Cd^2+^-EDTA-BSA (0.1 mg/mL) was dispensed on the special area of NC membrane, designated as the test line (T-line) by using the automatic dispenser with volume of 1 μL/cm. the NC membrane was dried at 37 ◦C for 24 h. The sample pad was pretreated by 0.01 M pH 7.2 PBS buffer containing 0.5% (w/v) BSA and 2% Triton X-100. The mAb-AuNPs was dispensed on the conjugate pad by using the automatic dispenser with volumes 2 μL/cm. After pads were dried at 37°C for 2 h, all ICSs components were assembled with 2 mm overlaps. These stacks were then cut into ICS strips and then placed in plastic housings. For detect hemoglobin: capture mAb (0.1 mg/mL) was dispensed on the special area of NC membrane, designated as the test line (T-line). The mAb-AuNPs was dispensed on the conjugate pad by using the automatic dispenser with volumes 2 μL/cm. Other steps were same as procedure of AuNPs ICSs for Cd^2+^.

**The performance of the AuNPs ICSs**

For detect Cd^2+^:60 μL series concentrations of Cd^2+^ solution were distilled in 50 nM EDTA-Na2 and then detected by using the developed AuNPs ICSs. After 15 min, the results of these ICSs were recorded on a simple camera and analysis by ImageJ software. For detect hemoglobin: 60 μL series concentrations of hemoglobin detected by using the developed AuNPs ICSs. After 15 min, the results of these ICSs were recorded on a simple camera and analysis by ImageJ software.

**Protocol of the ELISA to detecting Cd^2+^**

The protocol of the assay is as follow: each well of the microtiter plates was coated with 100 μL of Cd^2+^-EDTA-BSA at concentration of 0.5 μg/mL and incubated 3 h at 37℃C. Unbound coating antigen was removed from the plate with the PBST buffer (The plates were washed three times with phosphate-buffered saline (PBS: 137 mM NaCl, 3 mM KCl, and 10 mM sodium phosphate,pH 7.4) containing 0.05% Tween 20), and each well was blocked with 5% BSA in PBST at 37 ℃ for 1 h.The mixture solution of 50 μL mAb (1:10000) and 50 μL sample solution were added to each well, and incubated for 30 min at 37 ℃. After removing the unbound mAb with the PBS buffer, 100 μL of goat anti-mouse polyclonal labeled HRP conjugate was added and incubated at 37 ℃ for 30 min. The plate was washed three times using washing PBST to remove unbound the conjugate. TMB solution was then added at100 μL/wel to each well and incubated for15 min at room temperature. The reaction was terminated by 50 μL/well of 2 M H_2_SO_4_. The absorbance was measured at 450 nm using a microplate reader.

**Protocol of the ELISA to detecting hemoglobin**

The protocol of the assay is as follow: each well of the microtiter plates was coated with 100 μL of capture mAb with concentration of 0.5 μg/mL and incubated 3 h at 37℃. Unbound coating antigen was removed from the plate with the PBST buffer (The plates were washed three times with phosphate-buffered saline (PBS: 137 mM NaCl, 3 mM KCl, and 10 mM sodium phosphate, pH 7.4) containing 0.05% Tween 20.), and each well was blocked with 5% BSA in PBST at 37 ℃ for 1h. The mixture solution of 100 μL sample solution were added to each well, and incubated for 30 min at 37 ℃. After removing the unbound mAb with the PBS buffer, 100 μL of label mAb labeled HRP conjugate was added and incubated at 37 ℃ for 30 min. The plate was washed three times using washing PBST to remove unbound hemoglobin. TMB solution was then added at 100 μL/well to each well and incubated for10 min at room temperature. The reaction was terminated by 50 μL/well of 2 M H_2_SO_4_. The absorbance was measured at 450 nm using a microplate reader.

### Preparation of AgNPs

AgNO_3_ (90 mg) was dissolved in 500 mL of H_2_O and brought to boiling. A solution of 1% sodium citrate (10 mL) was added. The solution was kept on boiling for 1 h.

### Preparation of Au@AgNPs

For the first cycle, 30 μL of AA (100 mM), 40 μL of AgNO_3_ (100 mM), and 30 μL of NaOH (100 mM) were added to a beaker containing 10mL of as-prepared Au NPs (15 nm) at room temperature. The pH of the mixture was around 8.5. The reaction was continued for 30 mi. After a selected number of additions, the resulting particles were centrifuged at 6000 rpm for 20 min and redispersed in 10 mL of water.


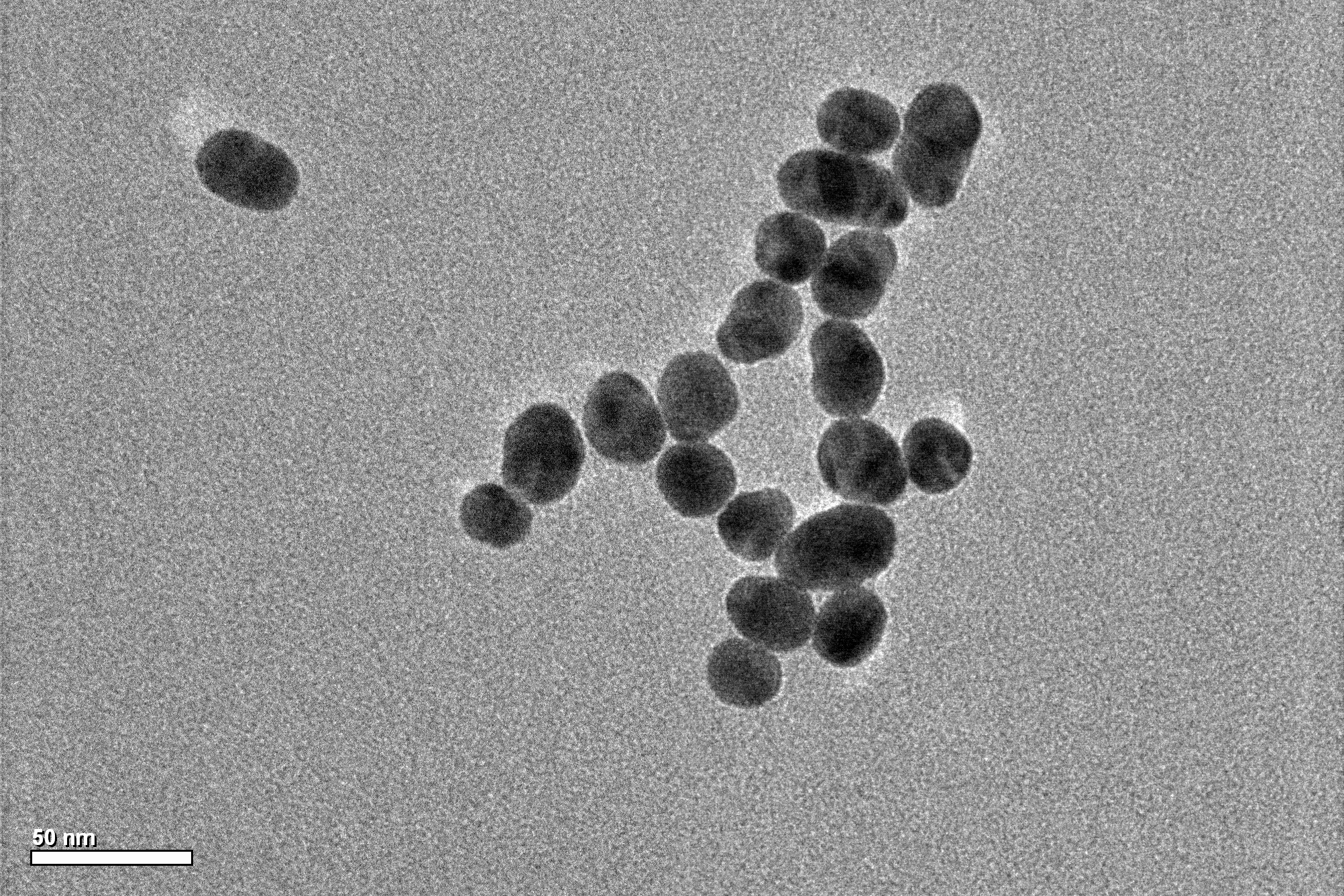


**Fig. S1.** TEM images of AuNPs


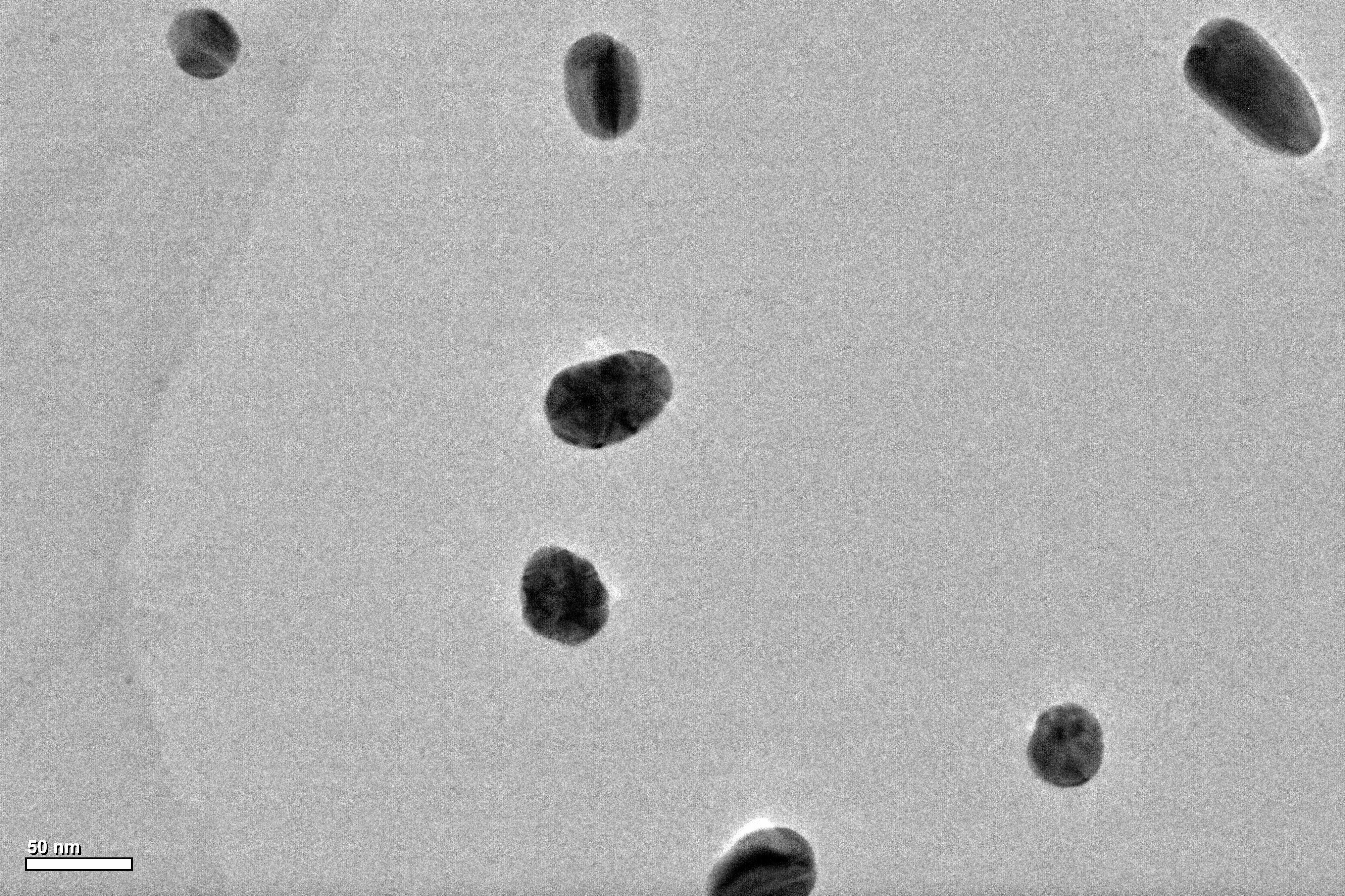


**Fig. S2.** TEM images of AgNPs


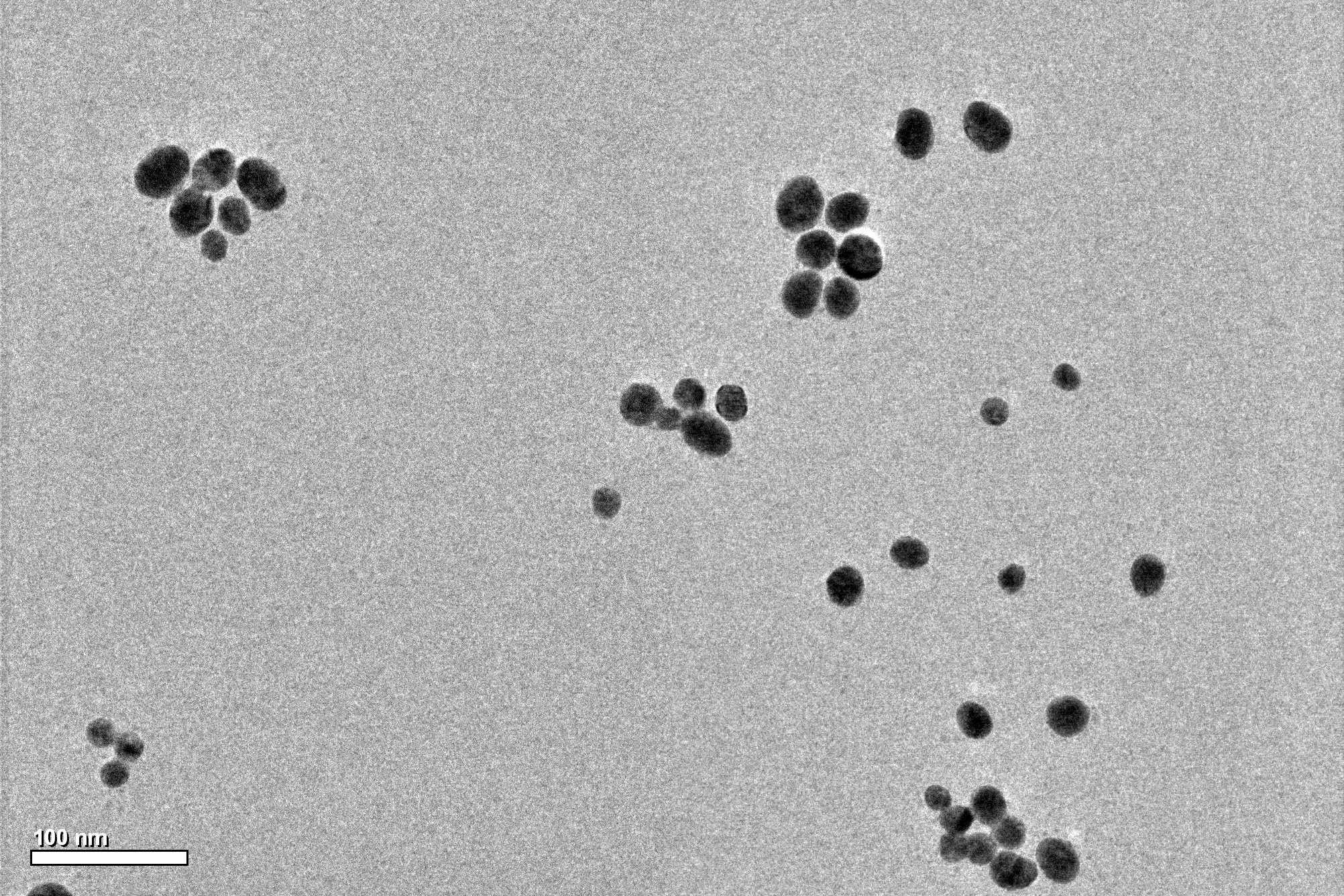


**Fig. S3.** TEM images of Au@AgNPs


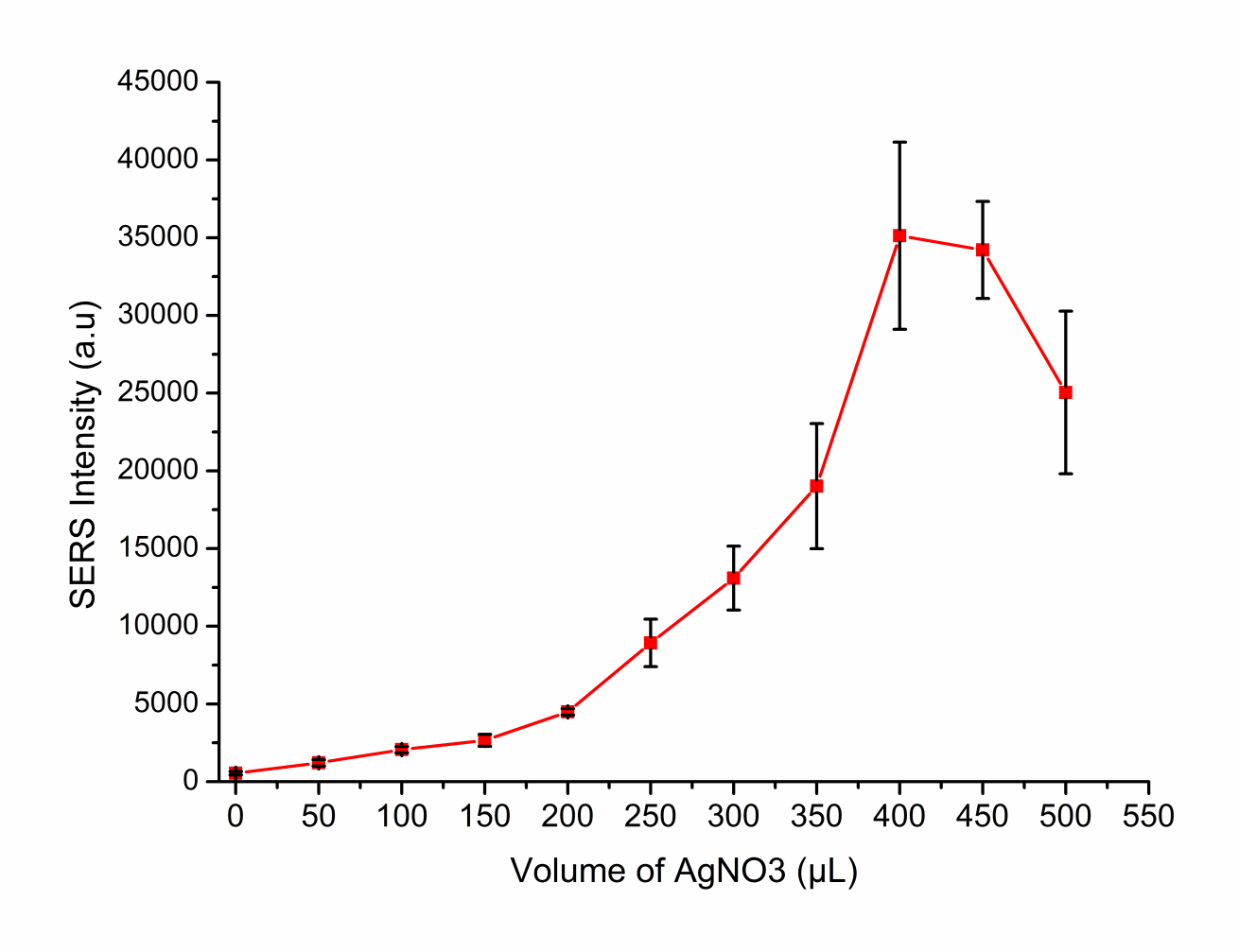


**Fig. S4.** Optimization of the volume of AgNO_3_ coated on AuNFs.


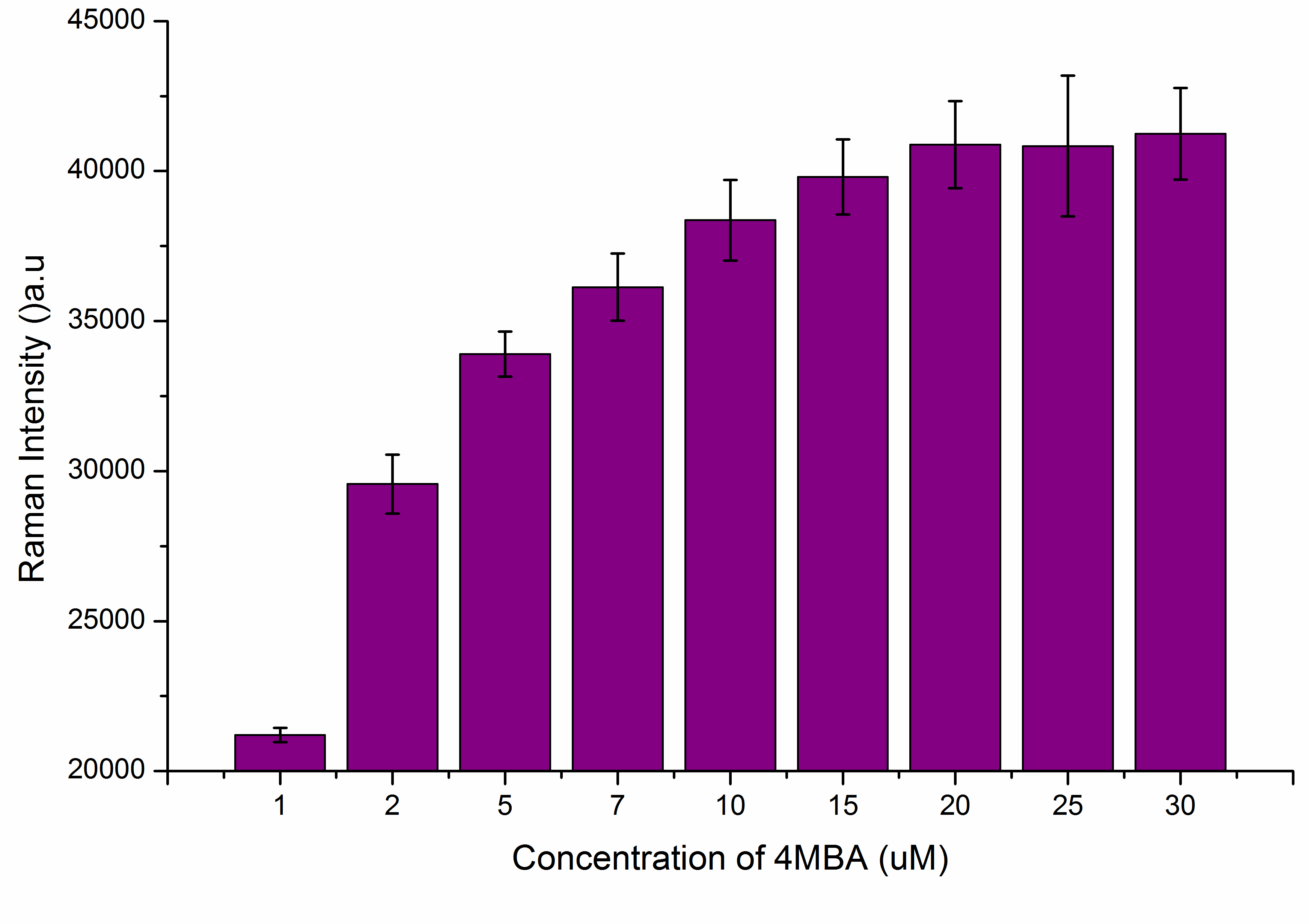


**Fig. S5.** Optimization of the concentration of 4MBA labeled on RSAu@AgNPs.


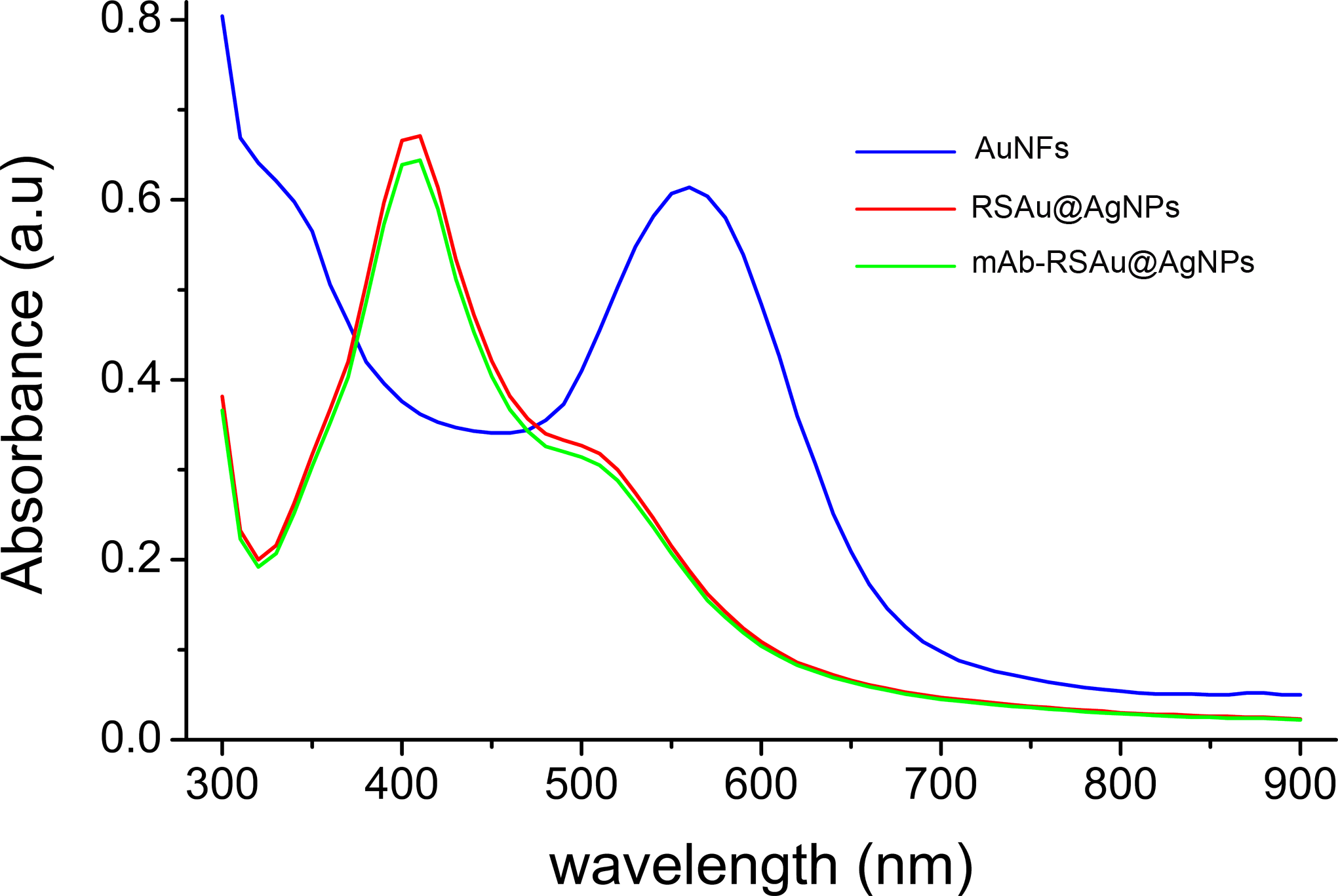


**Fig. S6.** The emission spectra of AuNFs, RSAu@AgNFs and mAb-RSAu@AgNFs.


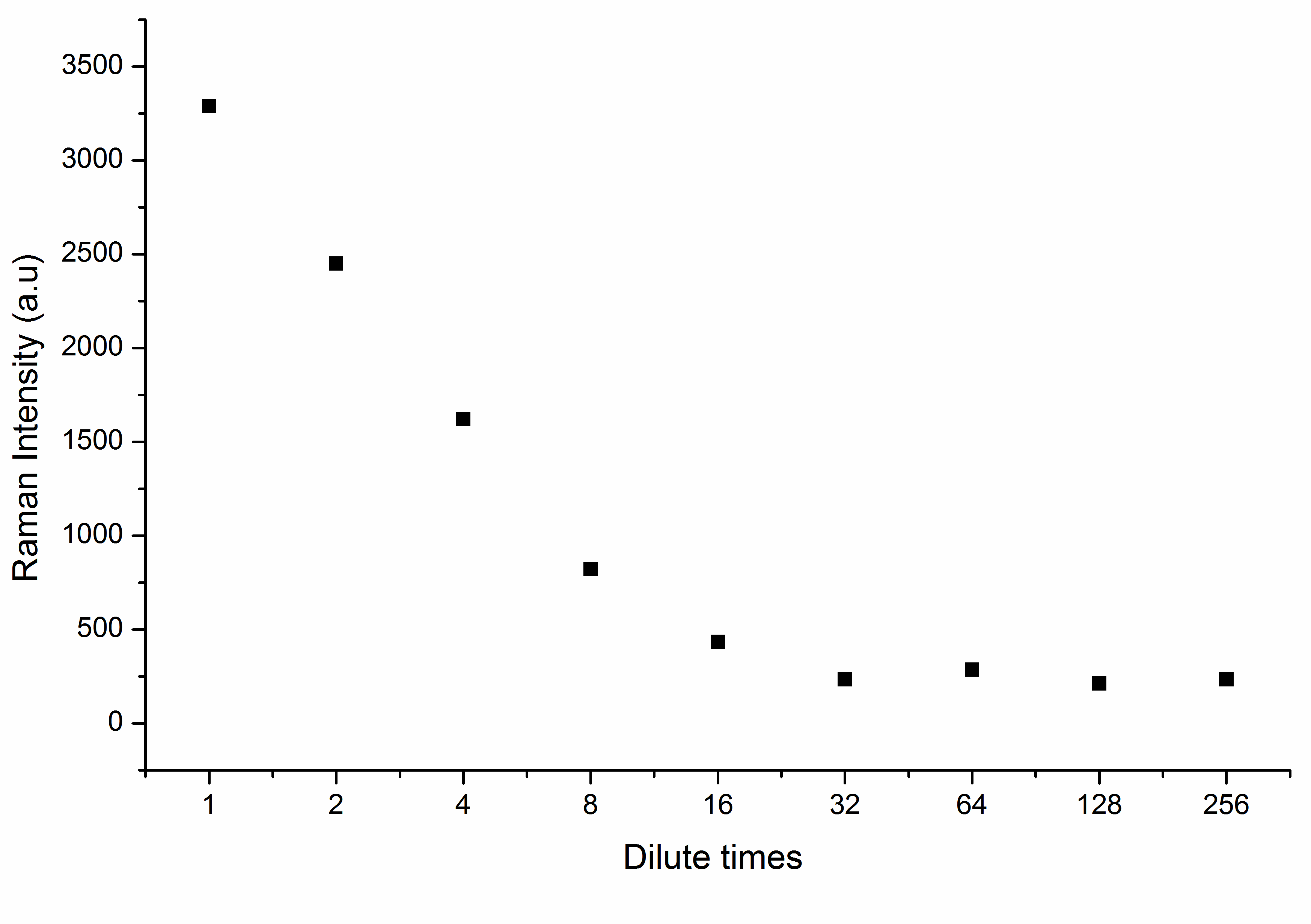


**Fig. S7.** Optimization of the dilute time mAb-RSAu@AgNPs-4MBA of the SERS ICSs for detect haemoglobin.


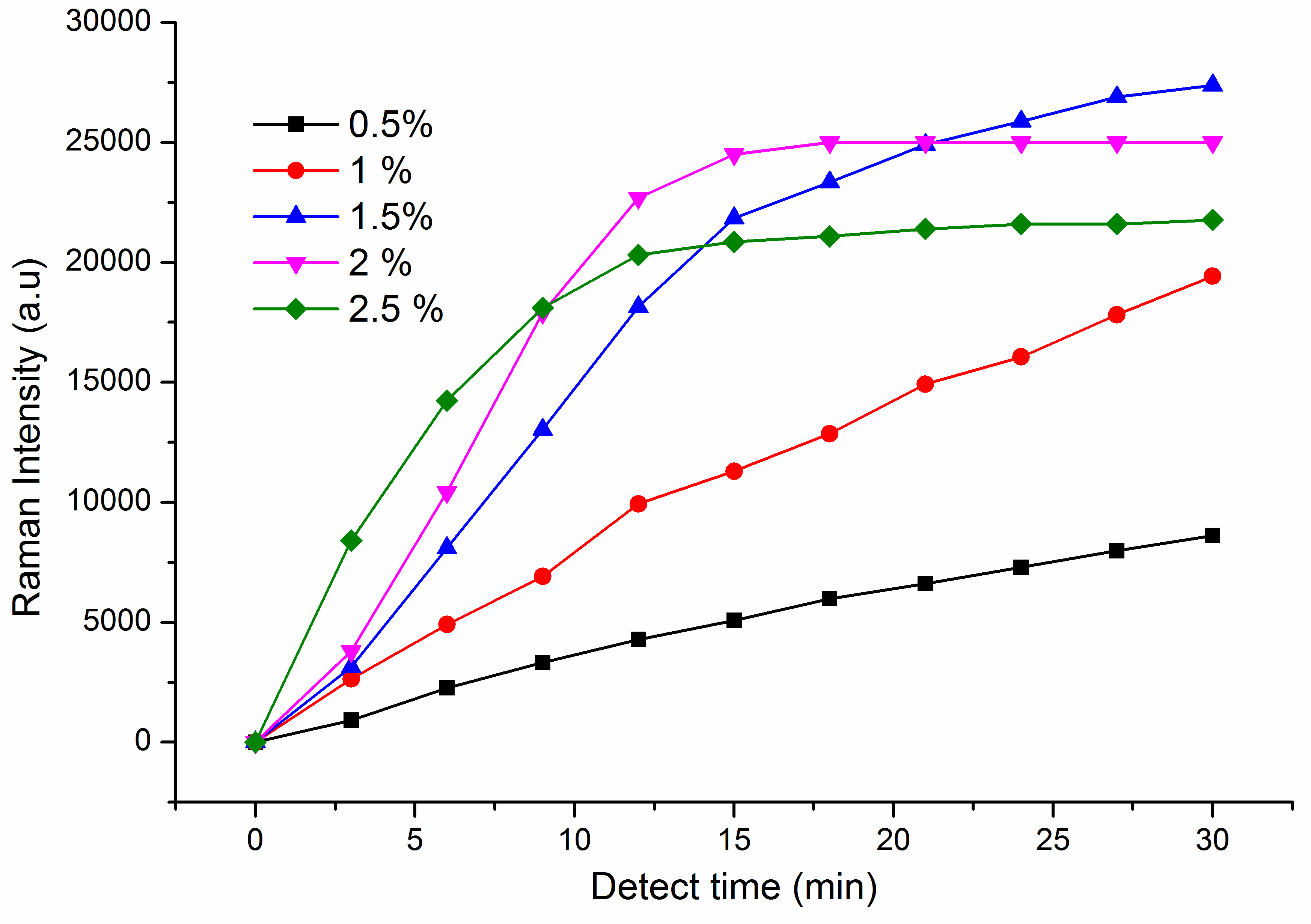


**Fig. S8.** Optimize concentration of triton-X 100 in sample pad treatment agent.


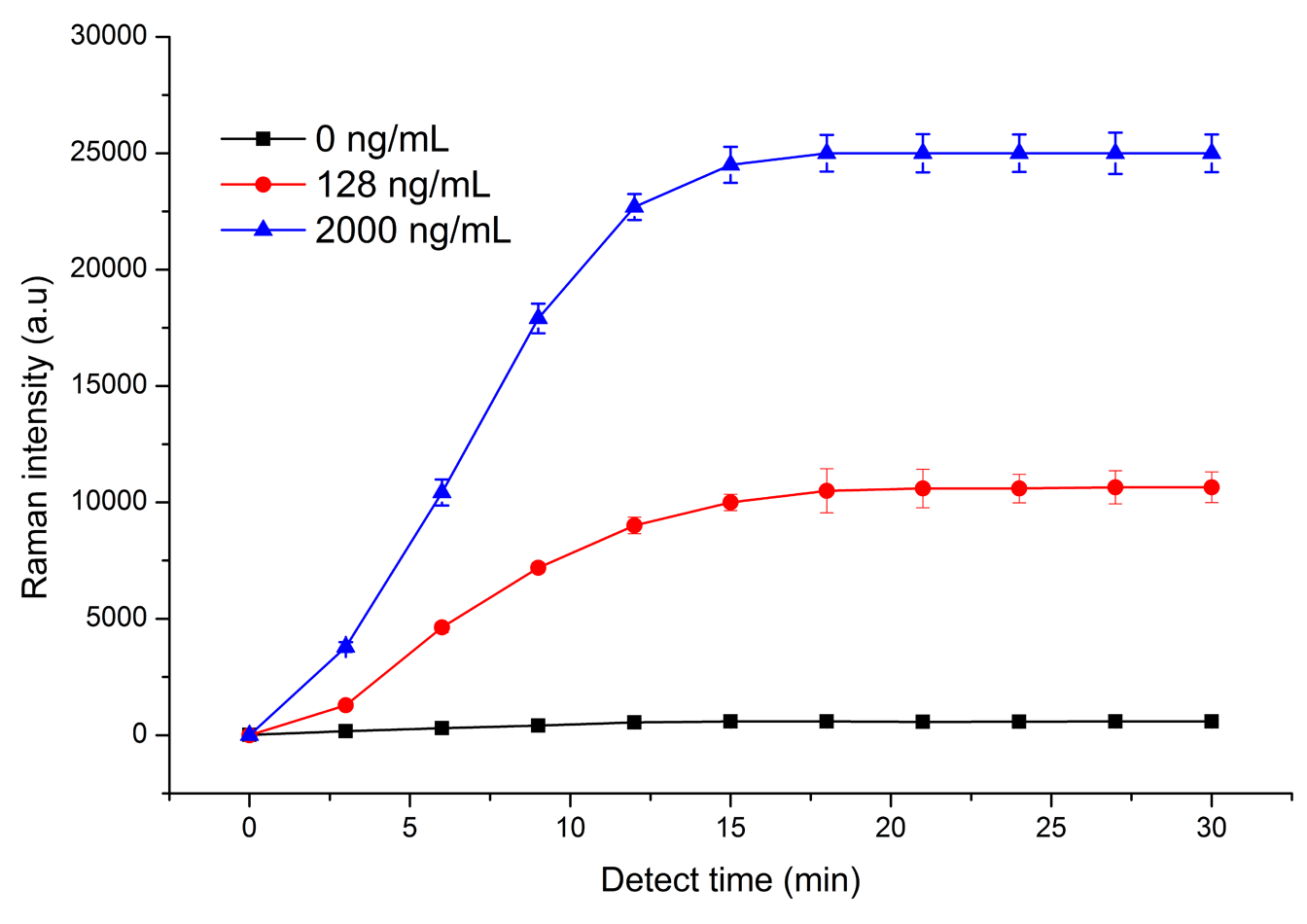


**Fig. S9.** Optimization of the SERS ICSs detection time for detect haemoglobin. 15 min was the optimal test time chosen for all following experiments.


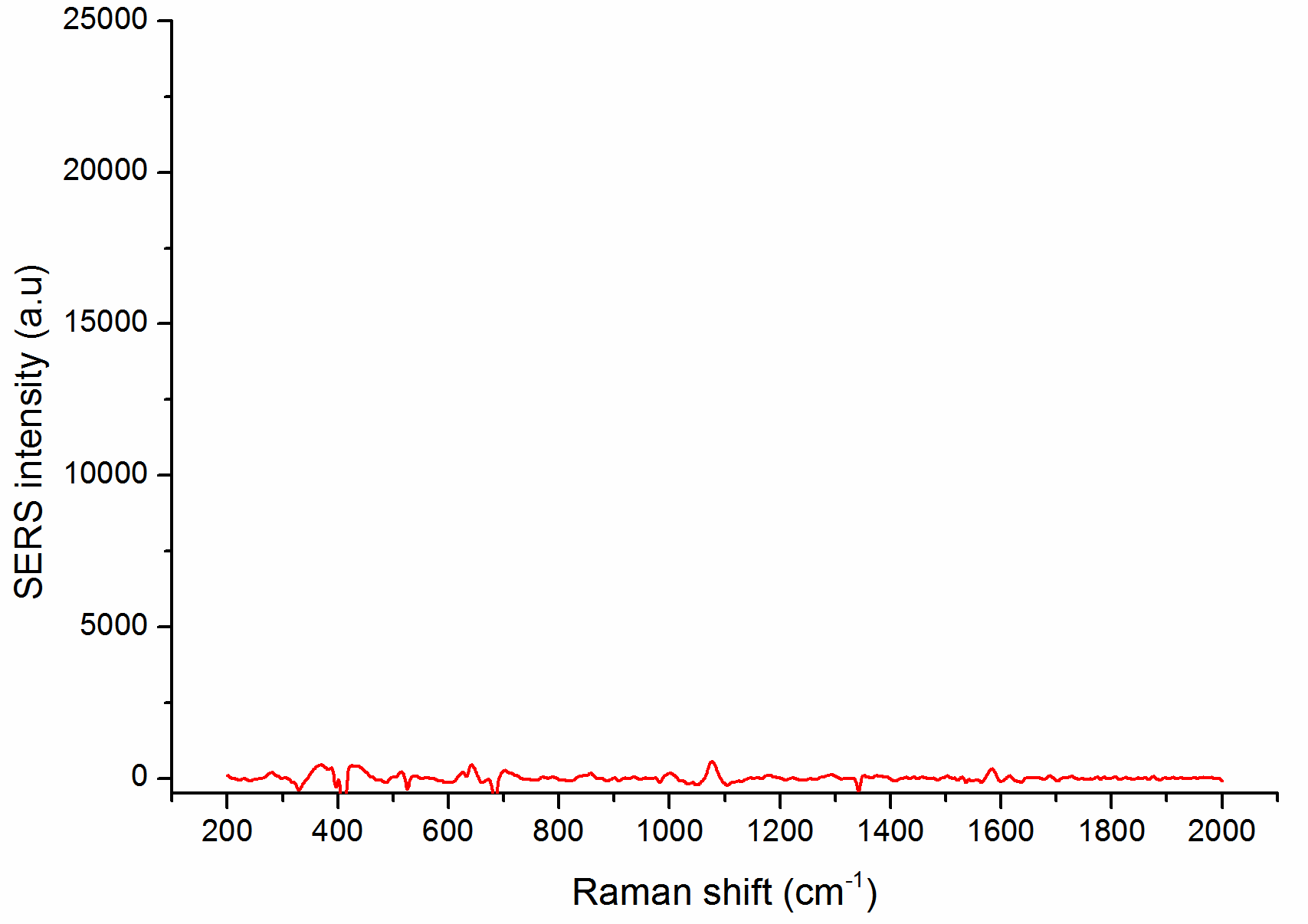


**Fig. S10.** Raman spectroscopy of SERS ICSs detect 0 ng/mL haemoglobin


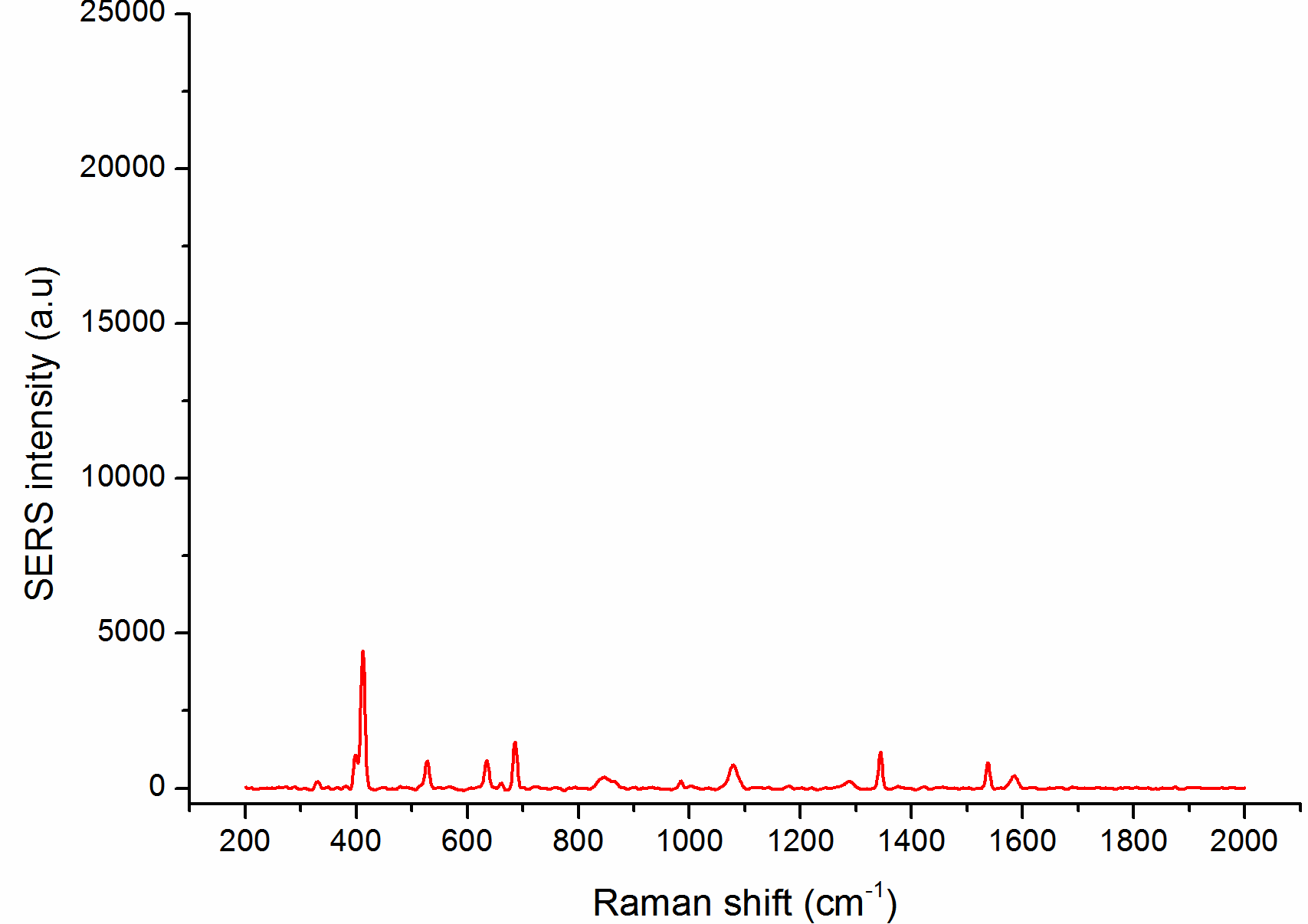


**Fig. S11.** Raman spectroscopy of SERS ICSs detect 15.6 ng/mL haemoglobin


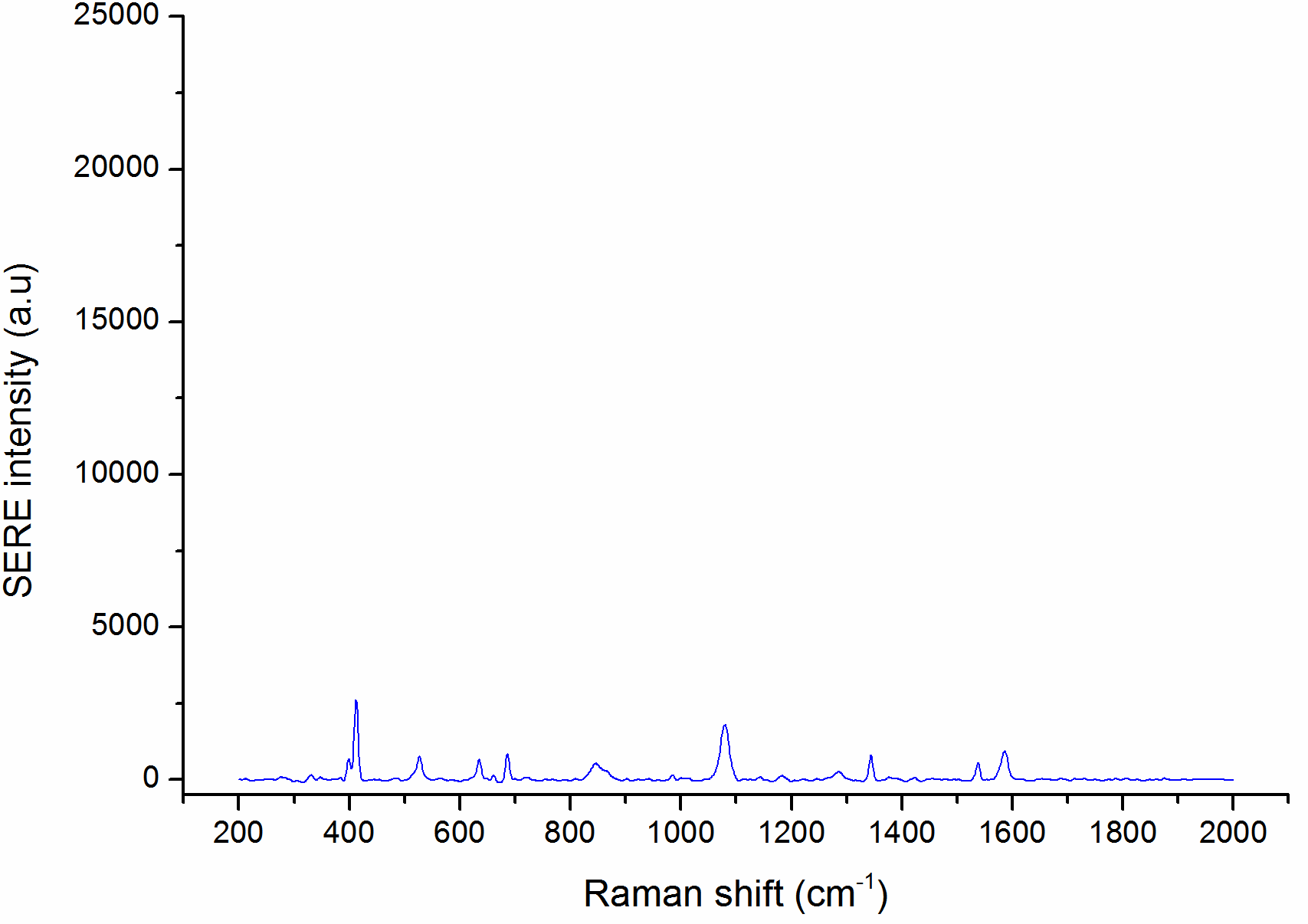


**Fig. S12.** Raman spectroscopy of SERS ICSs detect 31.3 ng/mL haemoglobin


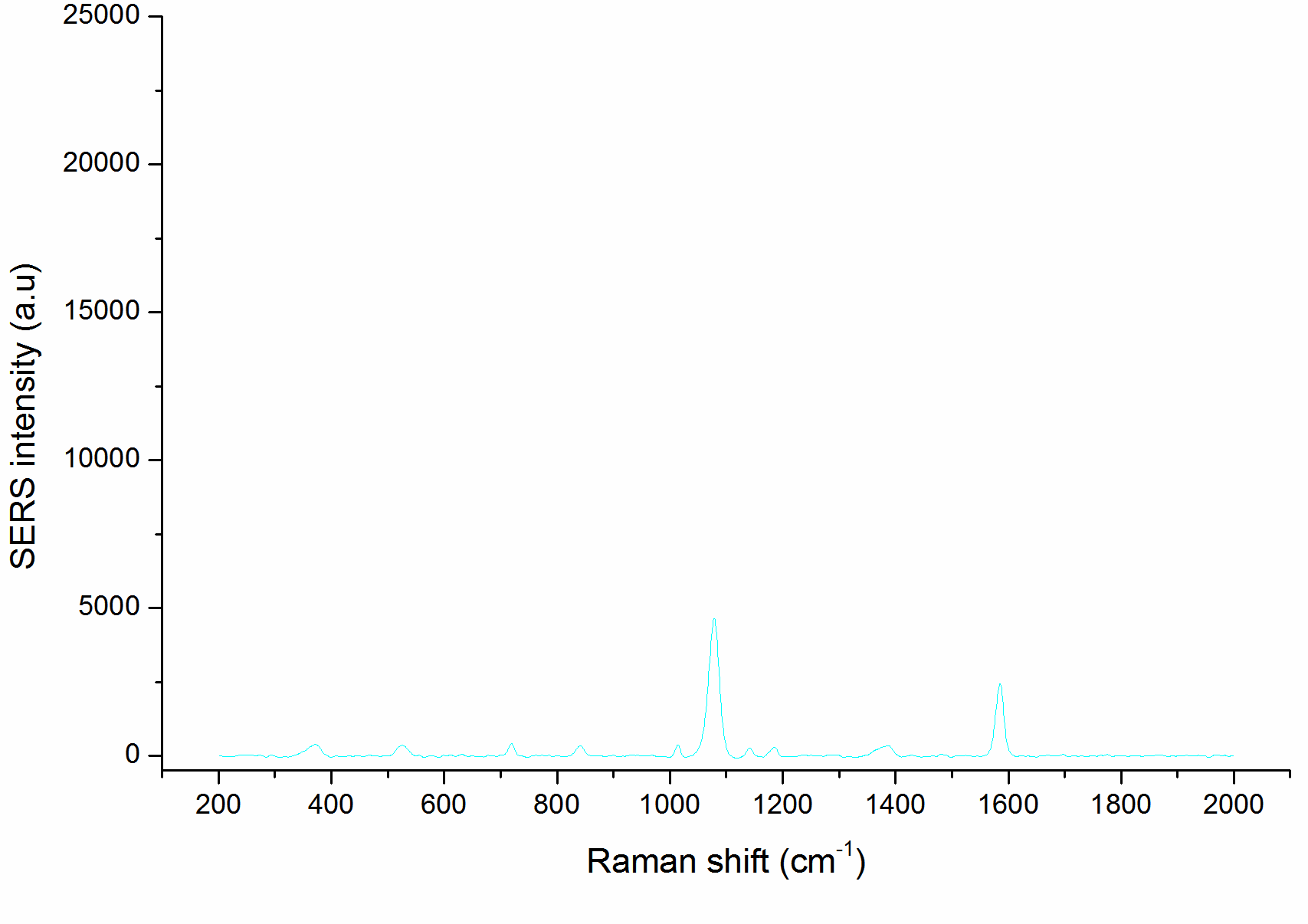


**Fig. S13.** Raman spectroscopy of SERS ICSs detect 62.5 ng/mL haemoglobin


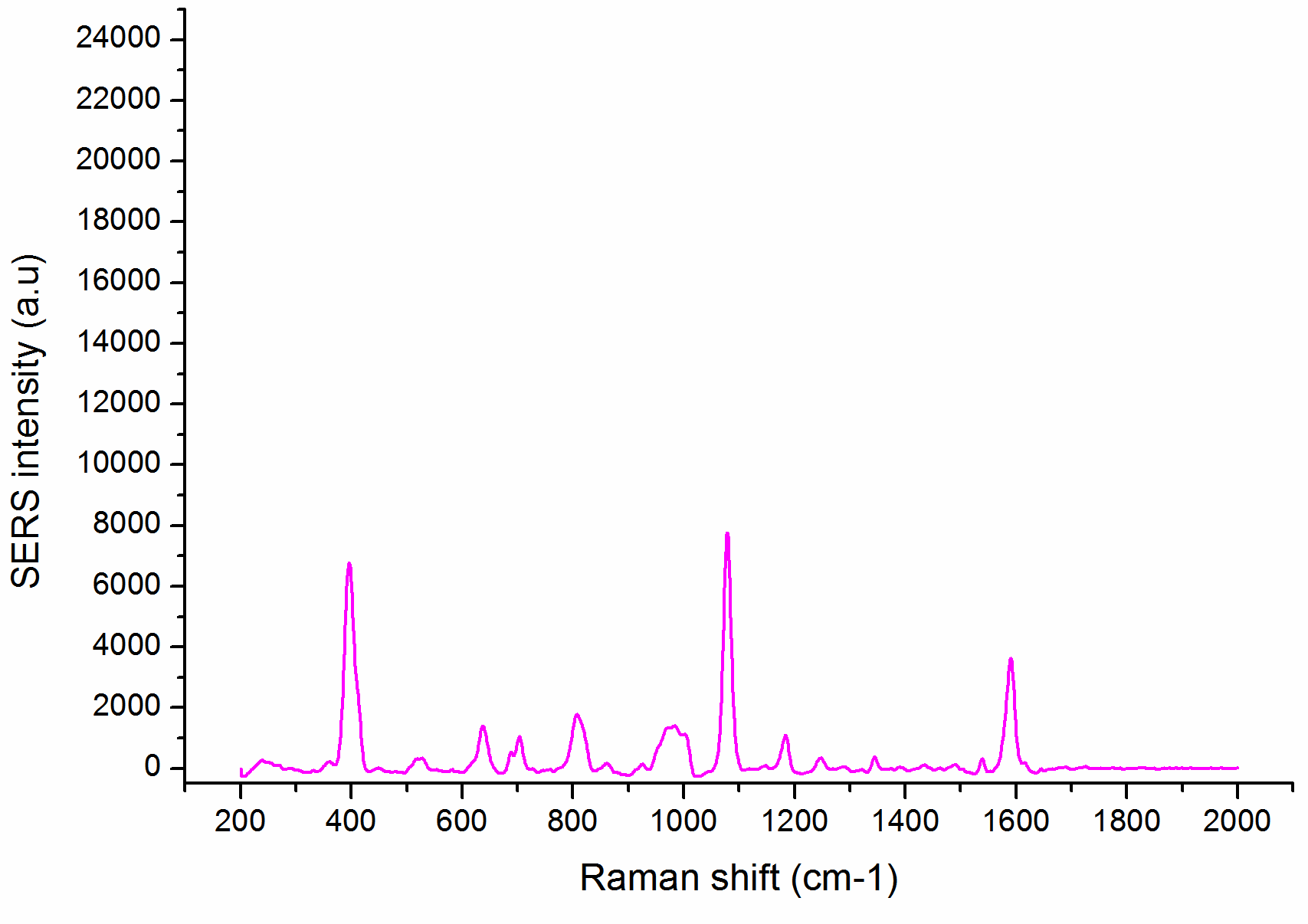


**Fig. S14.** Raman spectroscopy of SERS ICSs detect 125 ng/mL haemoglobin


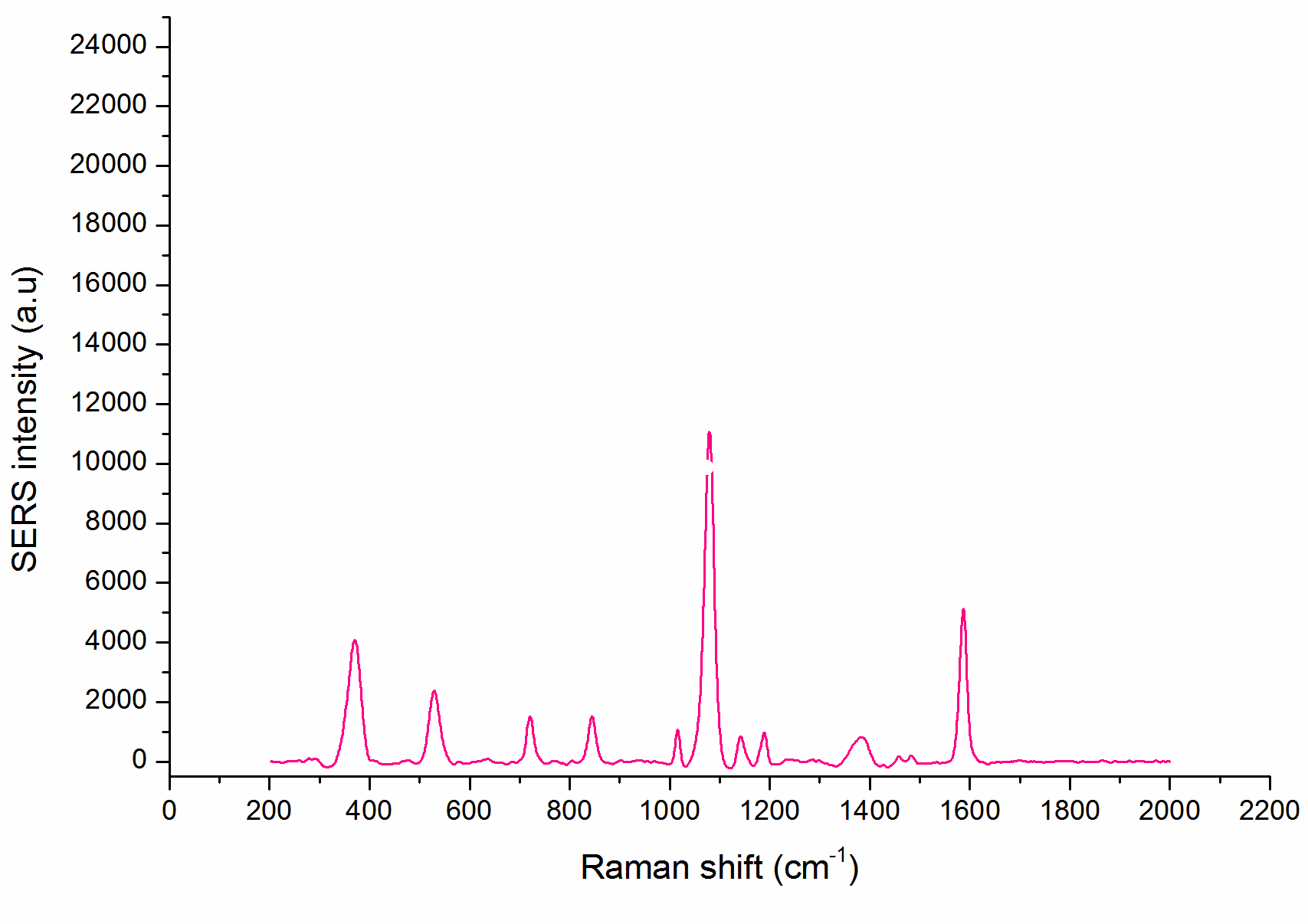


**Fig. S15.** Raman spectroscopy of SERS ICSs detect 250 ng/mL haemoglobin


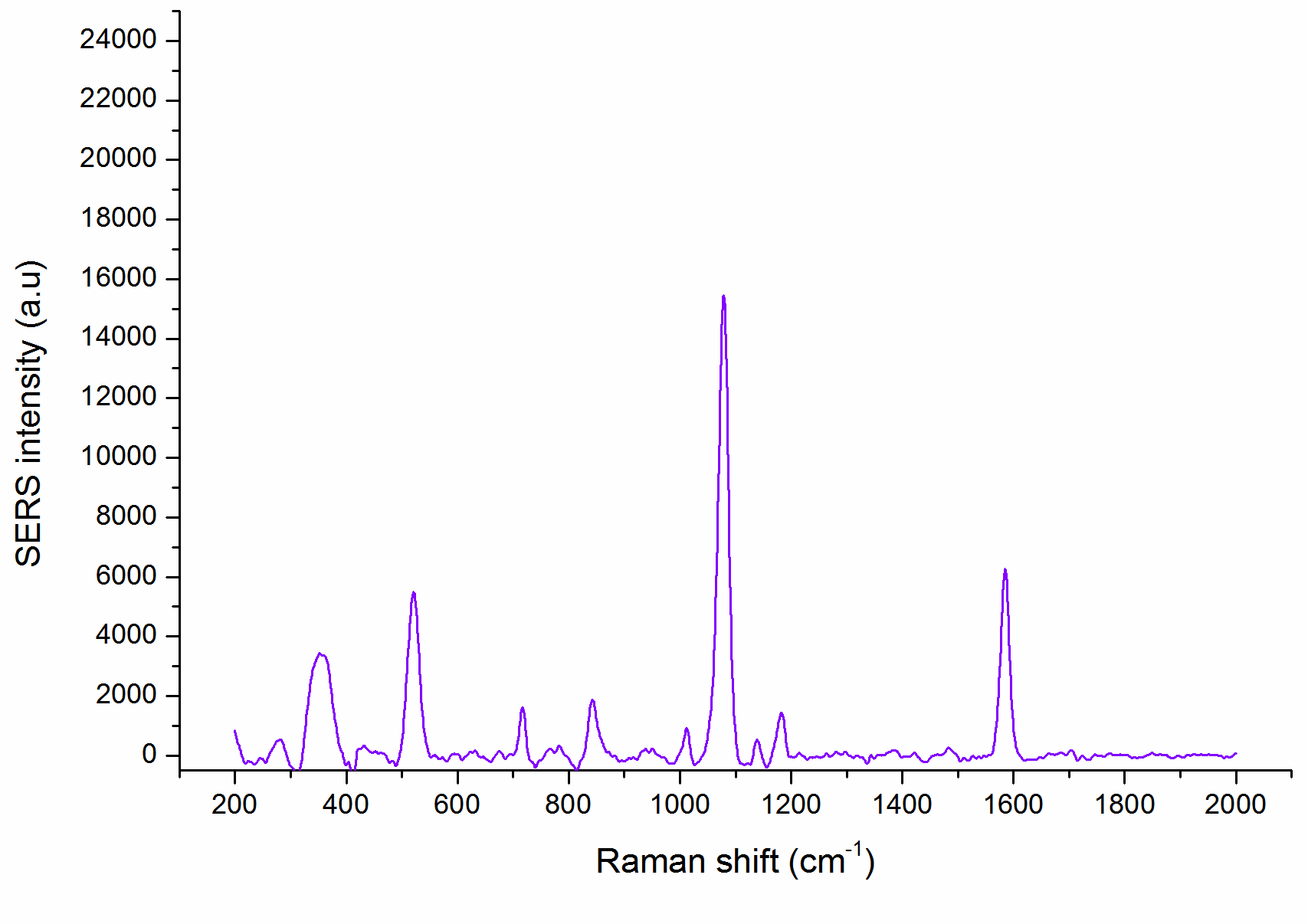


**Fig. S16.** Raman spectroscopy of SERS ICSs detect 500 ng/mL haemoglobin


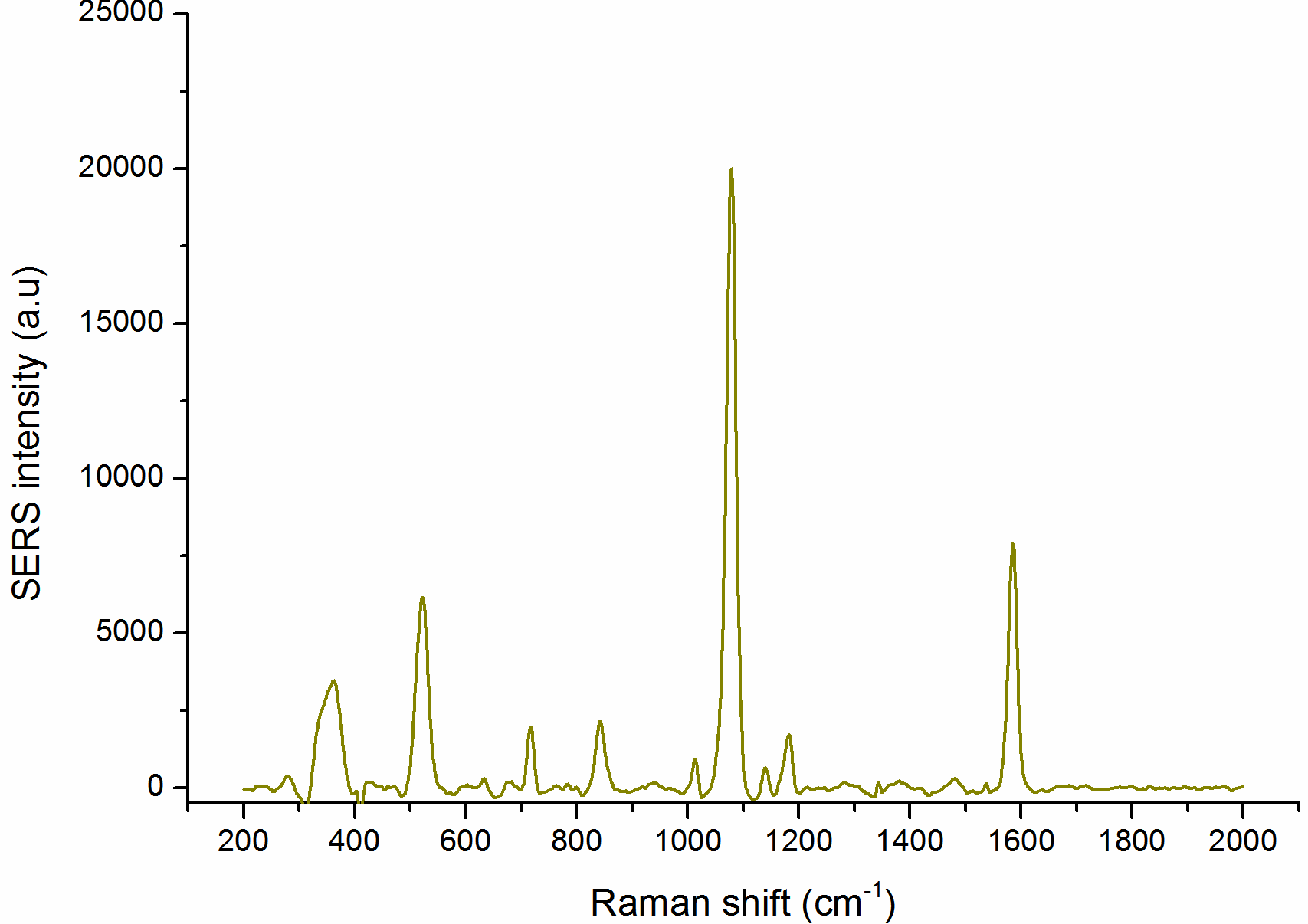


**Fig. S17.** Raman spectroscopy of SERS ICSs detect 1000 ng/mL haemoglobin


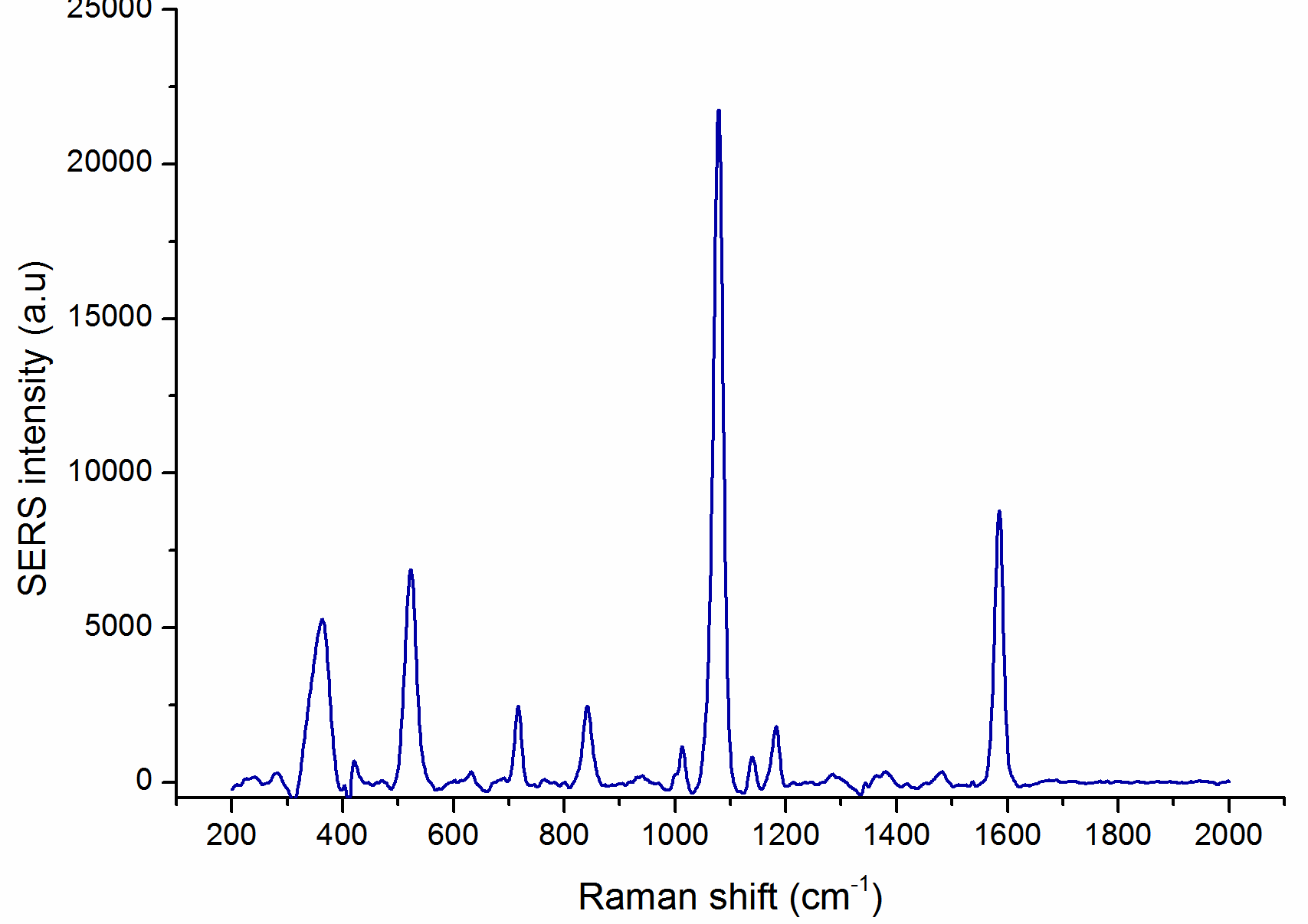


**Fig. S18.** Raman spectroscopy of SERS ICSs detect 2000 ng/mL haemoglobin


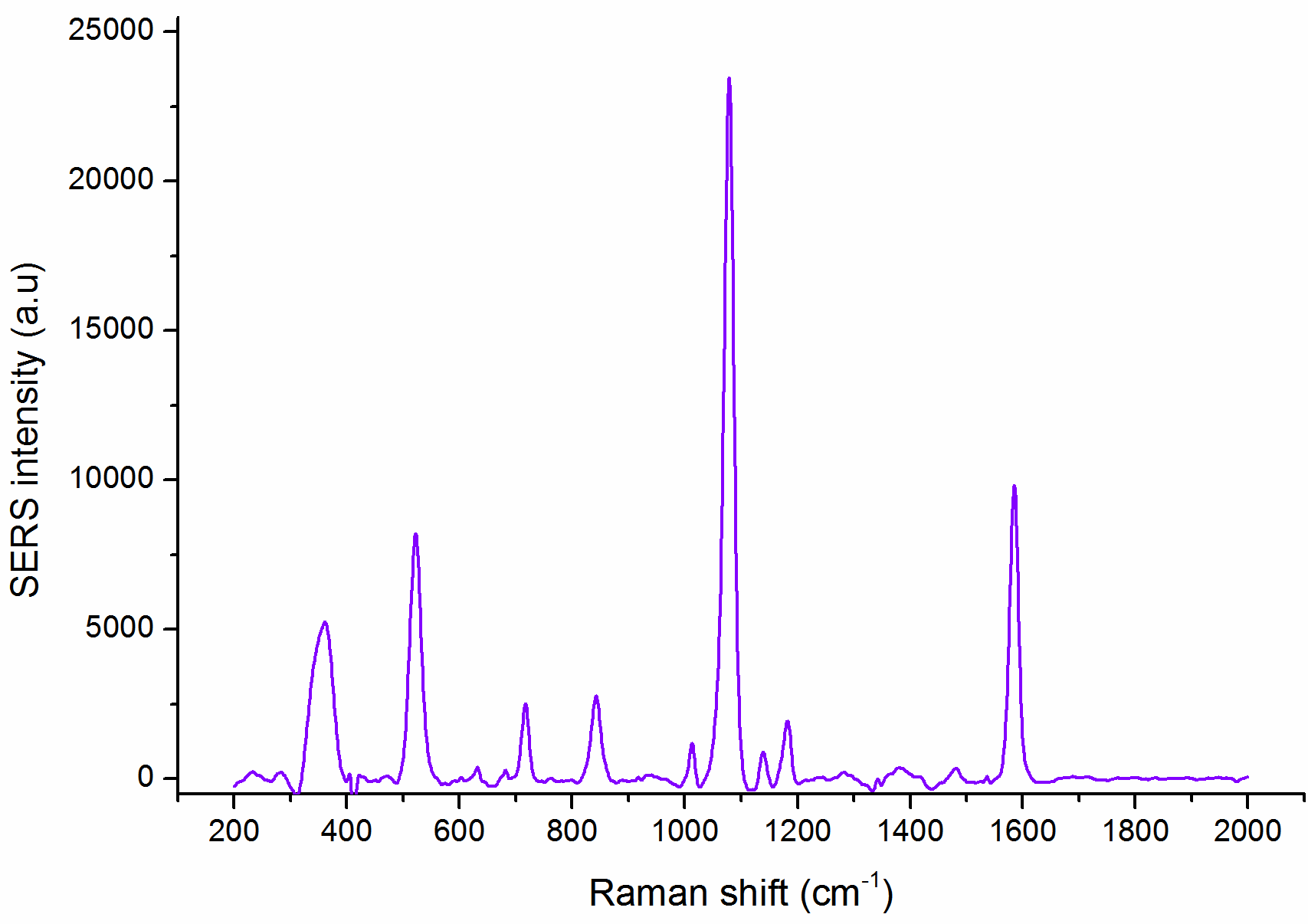


**Fig. S19.** Raman spectroscopy of SERS ICSs detect 4000 ng/mL haemoglobin


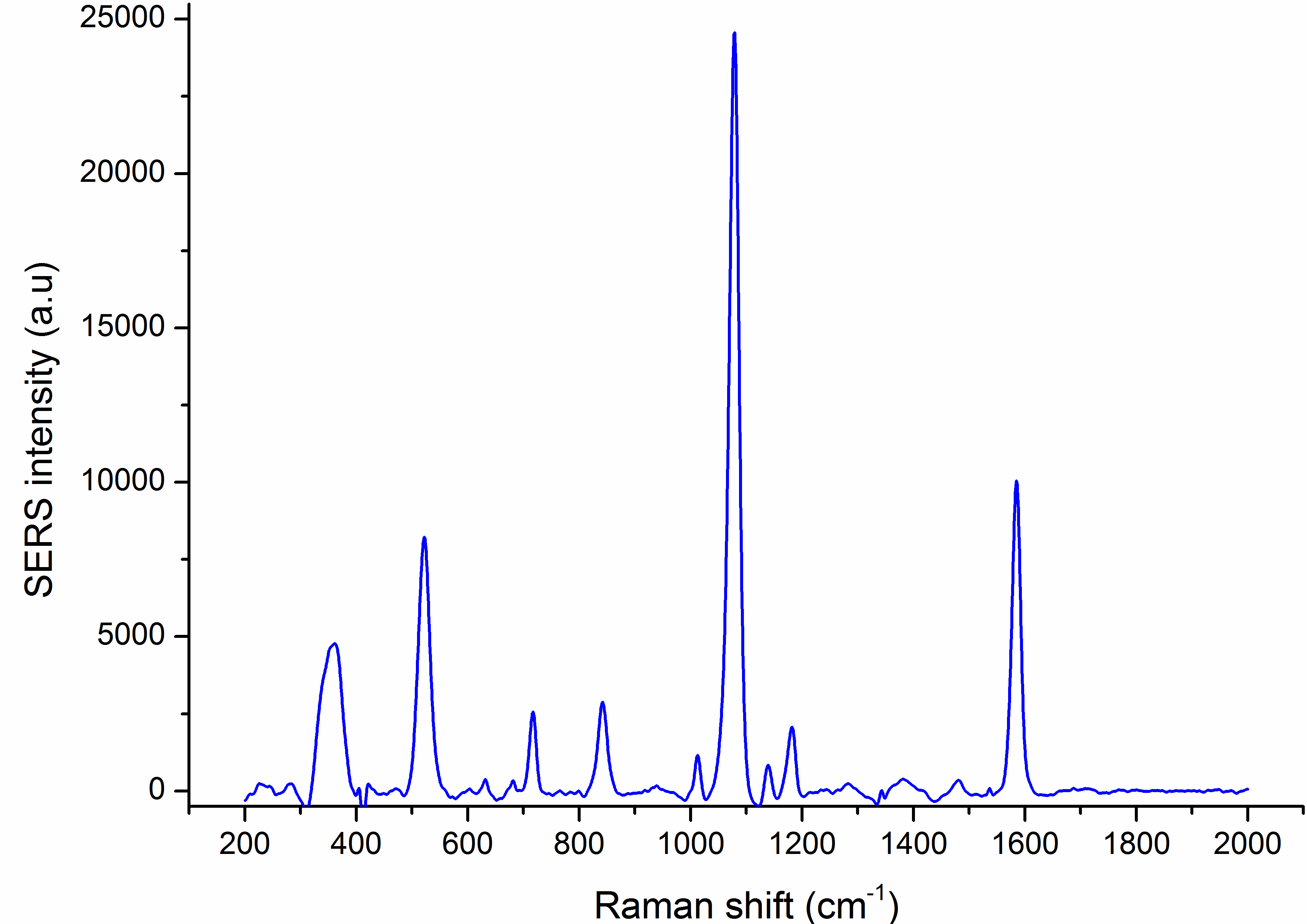


**Fig. S20.** Raman spectroscopy of SERS ICSs detect 8000 ng/mL haemoglobin


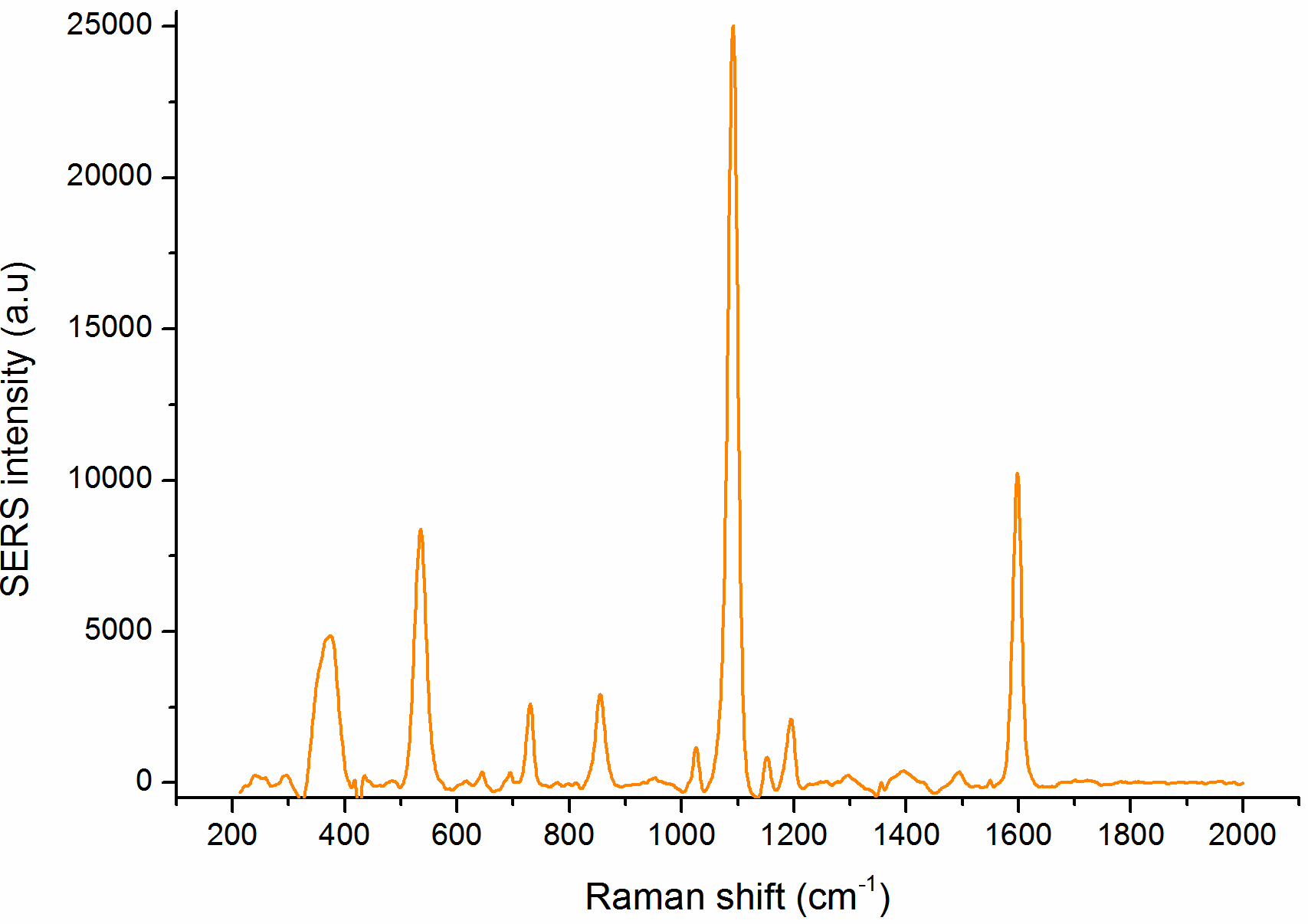


**Fig. S21.** Raman spectroscopy of SERS ICSs detect 16000 ng/mL haemoglobin

**Table S1.** Detailed Raman peaks of SERS ICSs for detect different concentration of haemoglobin

| Concentrations | 0 | 15.6 | 31.3 | 62.5 | 125 | 250 | 500 | 1000 | 2000 | 4000 | 8000 | 16000 |
| --- | --- | --- | --- | --- | --- | --- | --- | --- | --- | --- | --- | --- |
| Raman peaks (1077 cm^-1^) | 550 | 751 | 1744. | 4480 | 7720 | 10870 | 14947 | 18916 | 21760 | 24267 | 25131 | 25264 |
| S.D. | 18.19 | 42.50 | 75.66 | 4449.2 | 496.17 | 828 | 2773 | 546 | 604 | 1045 | 1537.6 | 1590 |
| Coefficient of variation (%)^c^ | 3.31 | 5.66 | 4.34 | 10.2 | 6.43 | 7.62 | 7.11 | 2.88 | 2.78 | 4.31 | 6.12 | 6.29 |


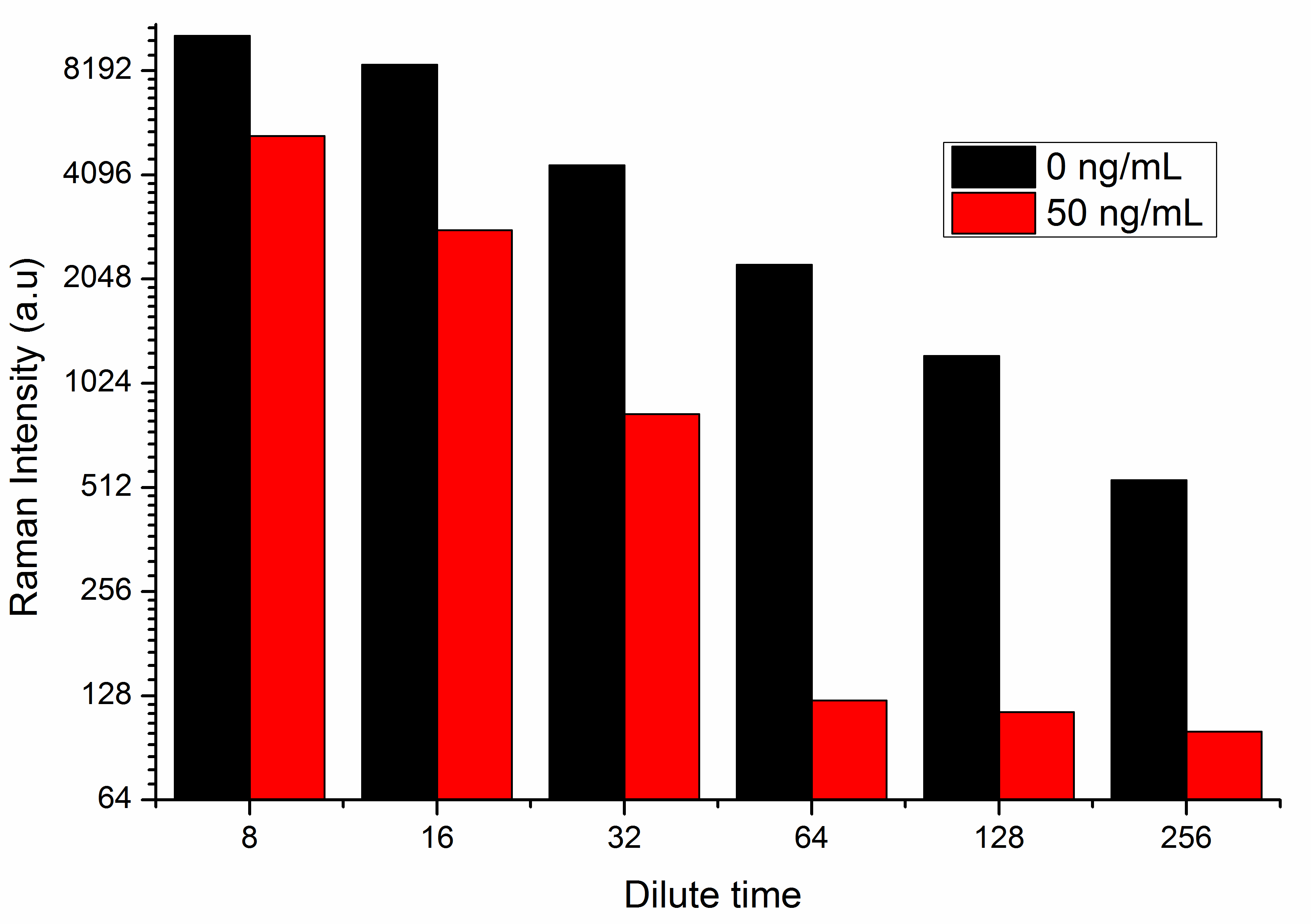


**Fig. S22.** Optimization of the concentration mAb-RSAu@AgNPs-4MBA of the SERS ICSs for detect Cd^2+^.

**Fig. S23.** Optimization of the SERS ICSs detection time for detect Cd^2+^. 15 min was the optimal test time chosen for all following experiments.


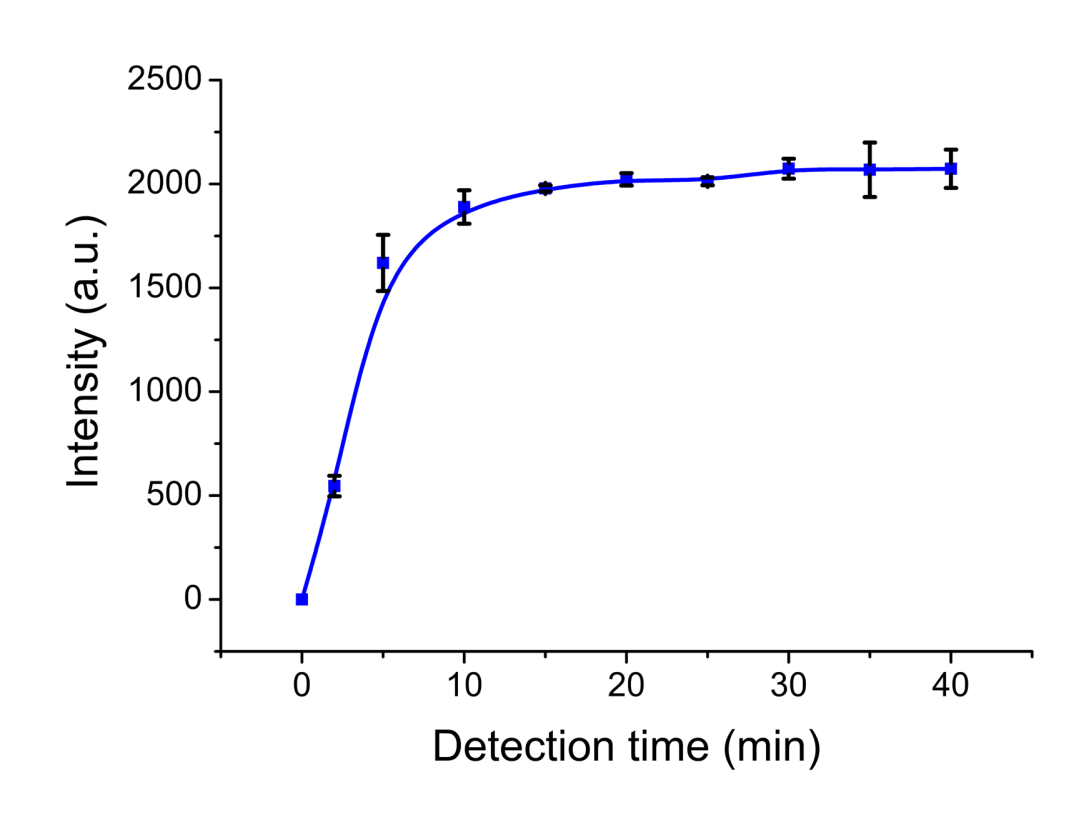

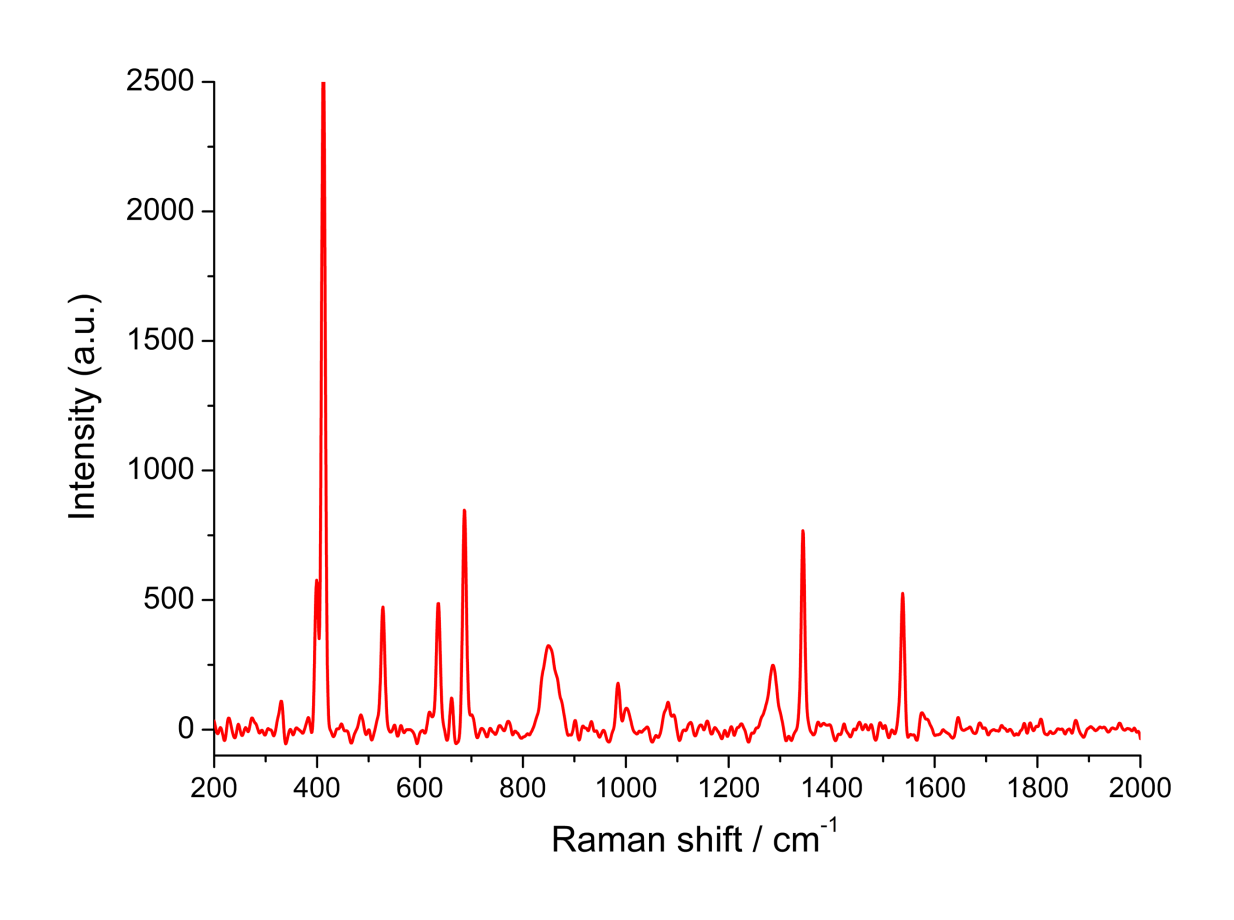


**Fig.S24** Raman spectroscopy of SERS ICSs detect 100 ng/mL Cd^2+^.


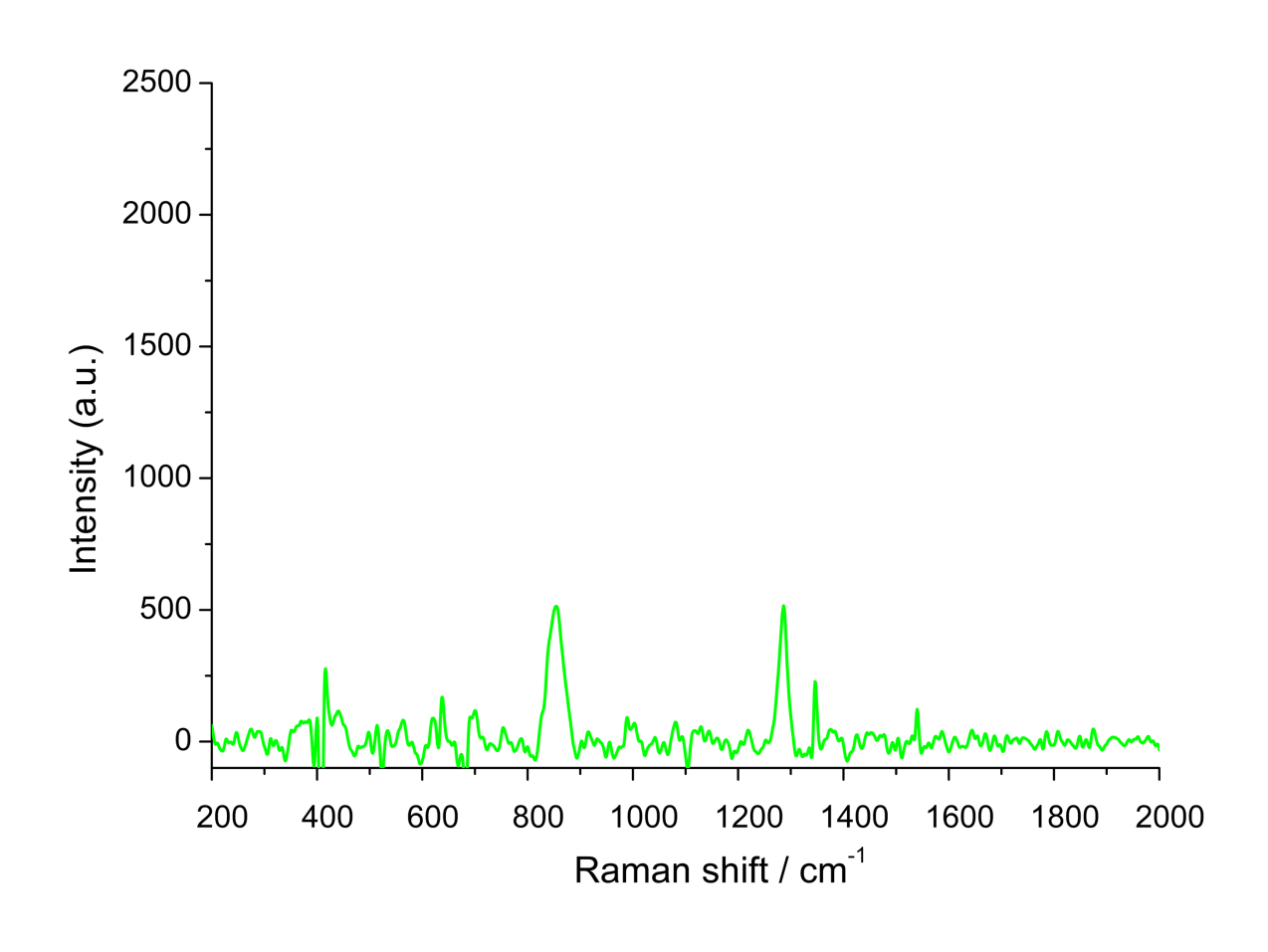


**Fig.S25** Raman spectroscopy of SERS ICSs detect 50 ng/mL Cd^2+^.


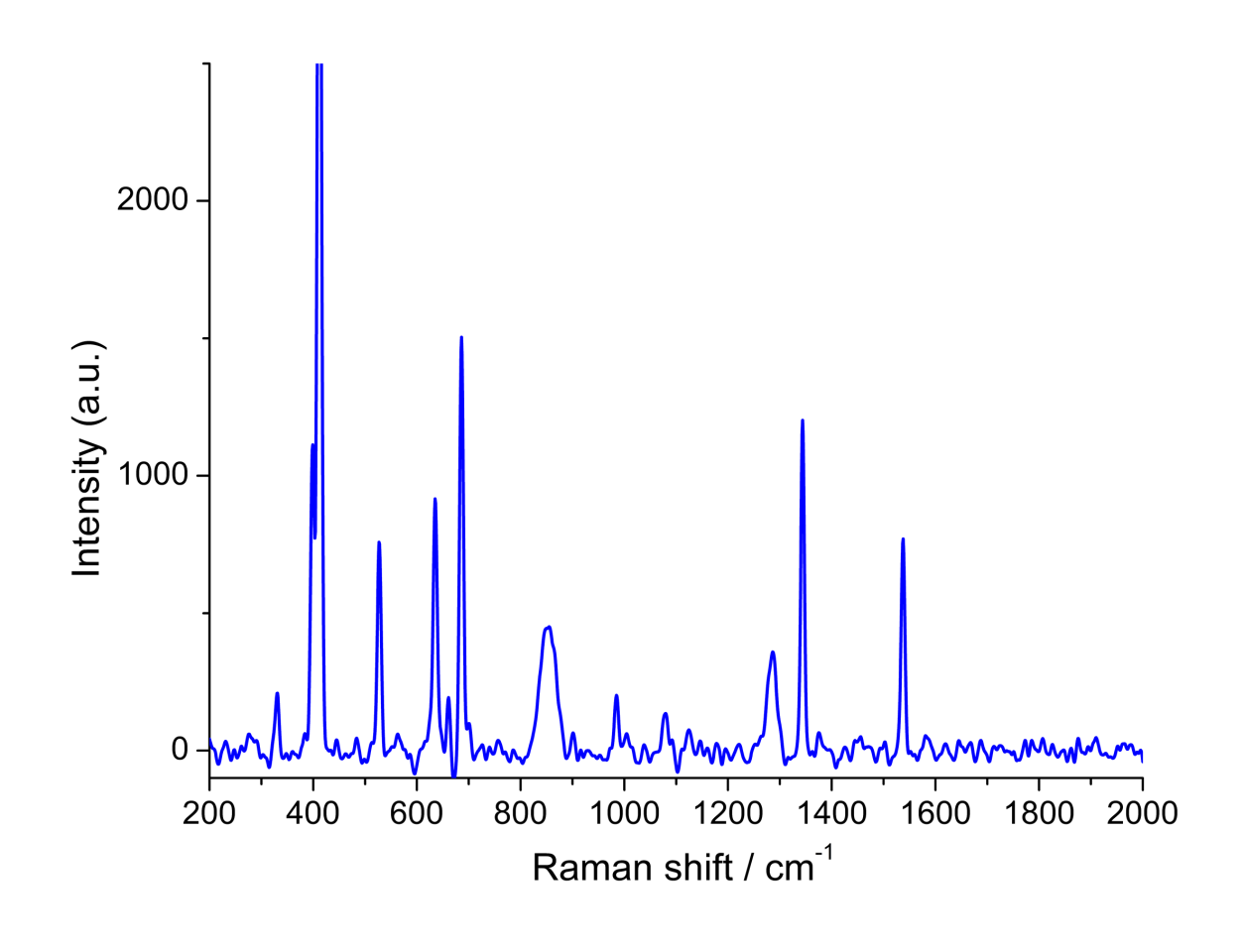


**Fig.S27** Raman spectroscopy of SERS ICSs detect 25 ng/mL Cd^2+^.


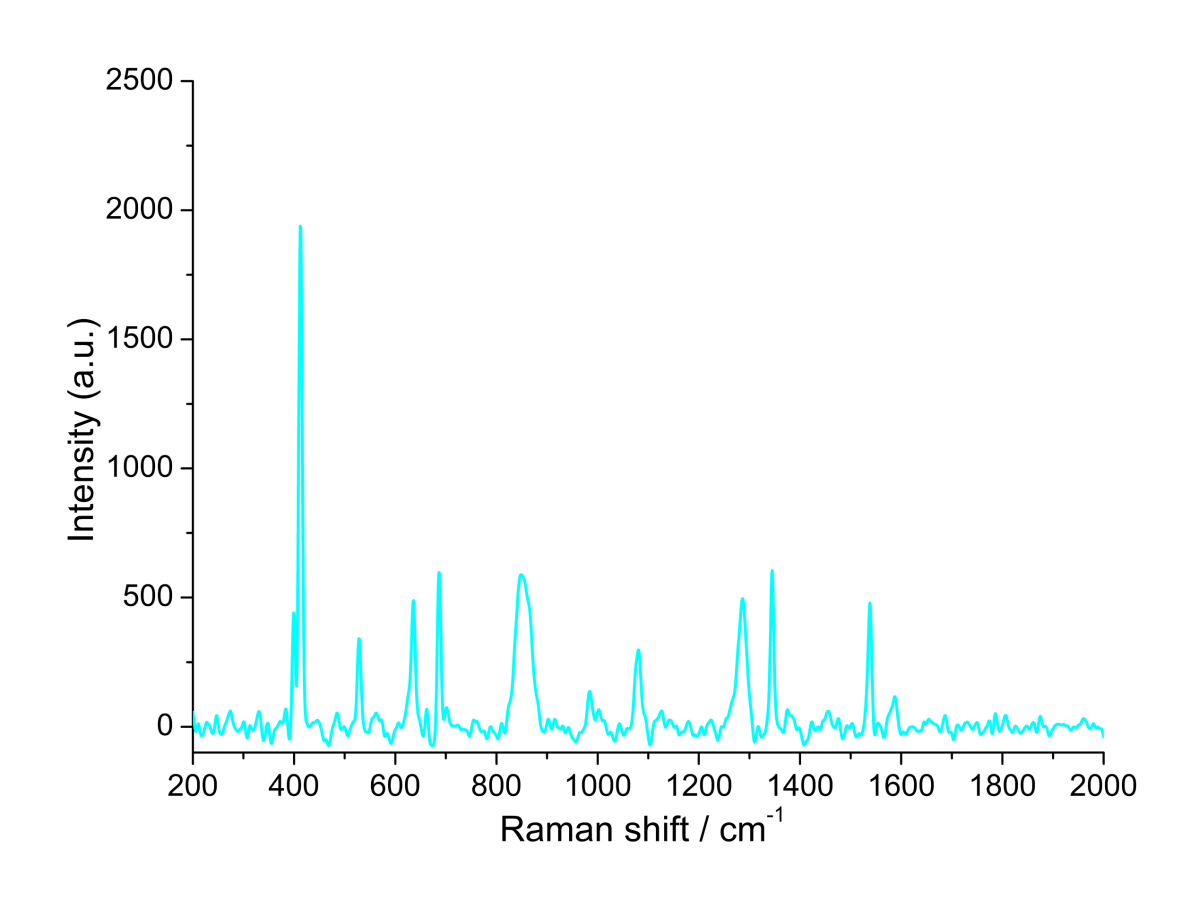


**Fig.S28** Raman spectroscopy of SERS ICSs detect 12.5 ng/mL Cd^2+^.


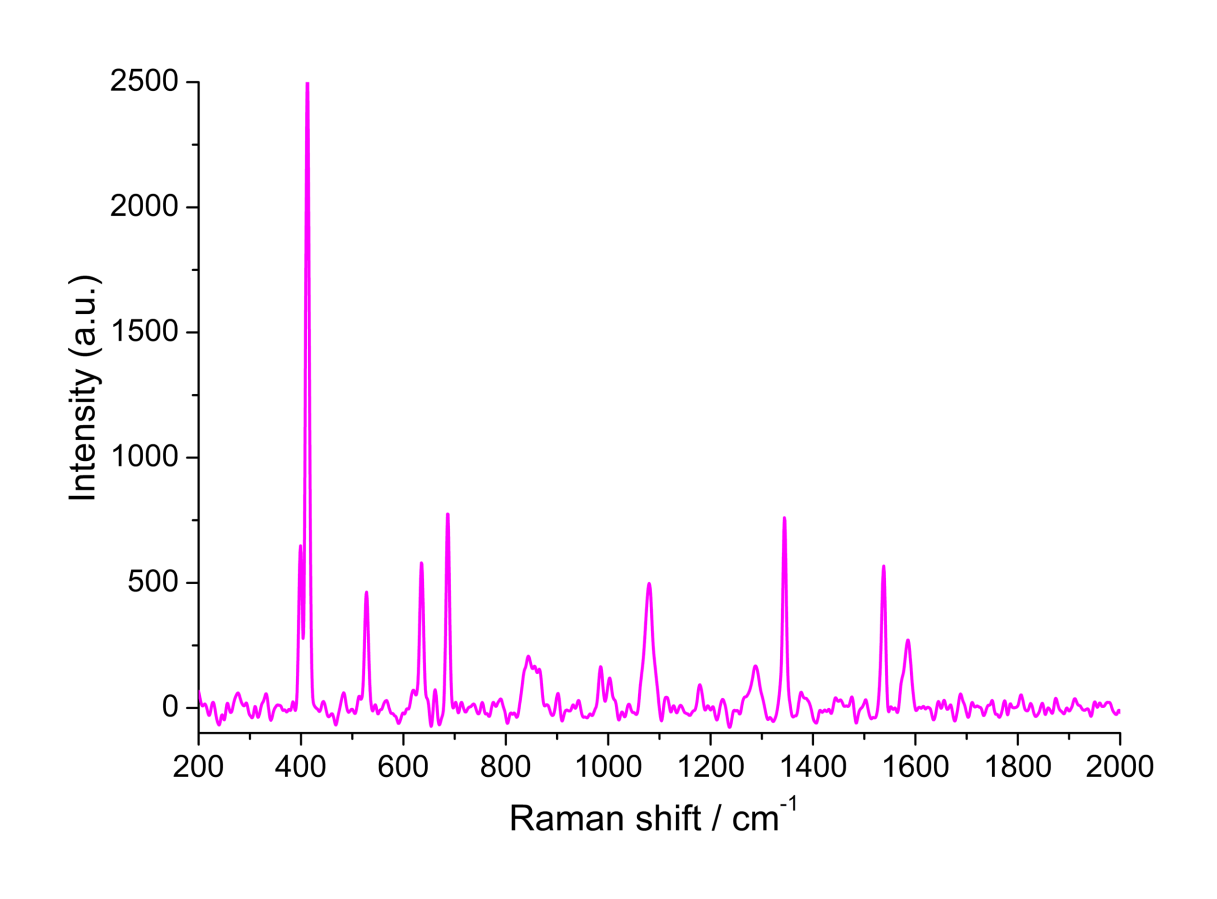


**Fig.S29** Raman spectroscopy of SERS ICSs detect 6.25 ng/mL Cd^2+^.


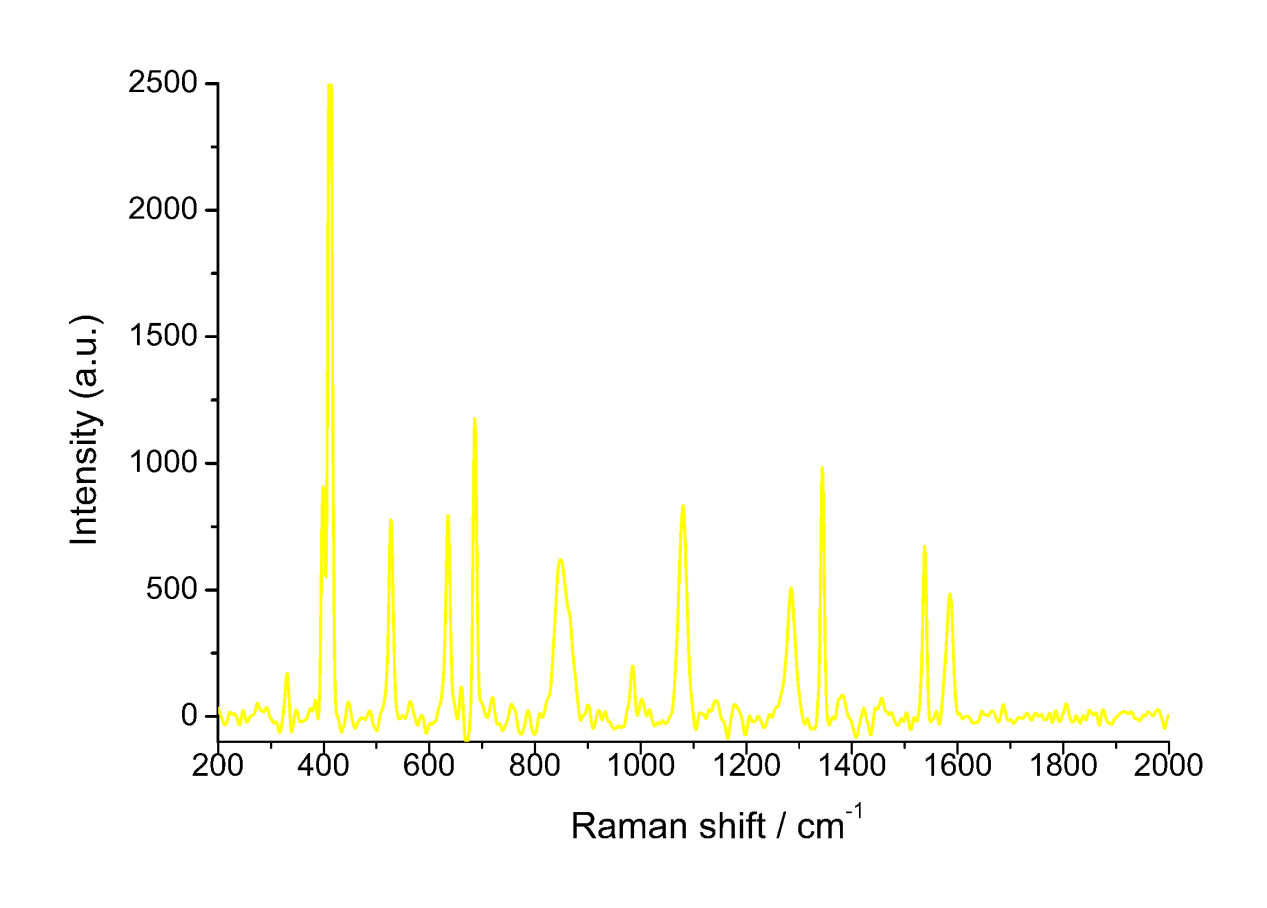


**Fig.S30** Raman spectroscopy of SERS ICSs detect 3.125 ng/mL Cd^2+^.


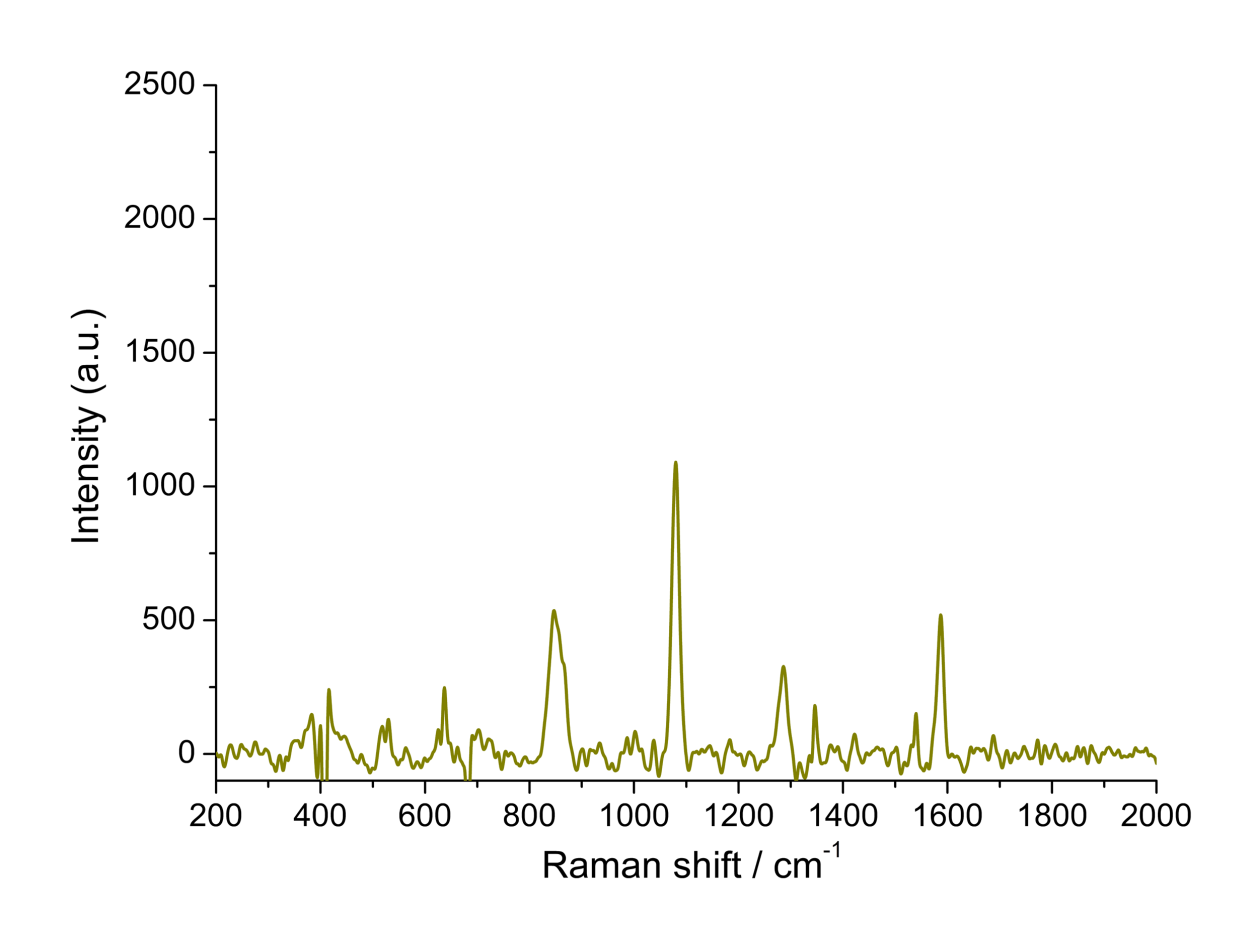


**Fig.S31** Raman spectroscopy of SERS ICSs detect 1.56 ng/mL Cd^2+^.


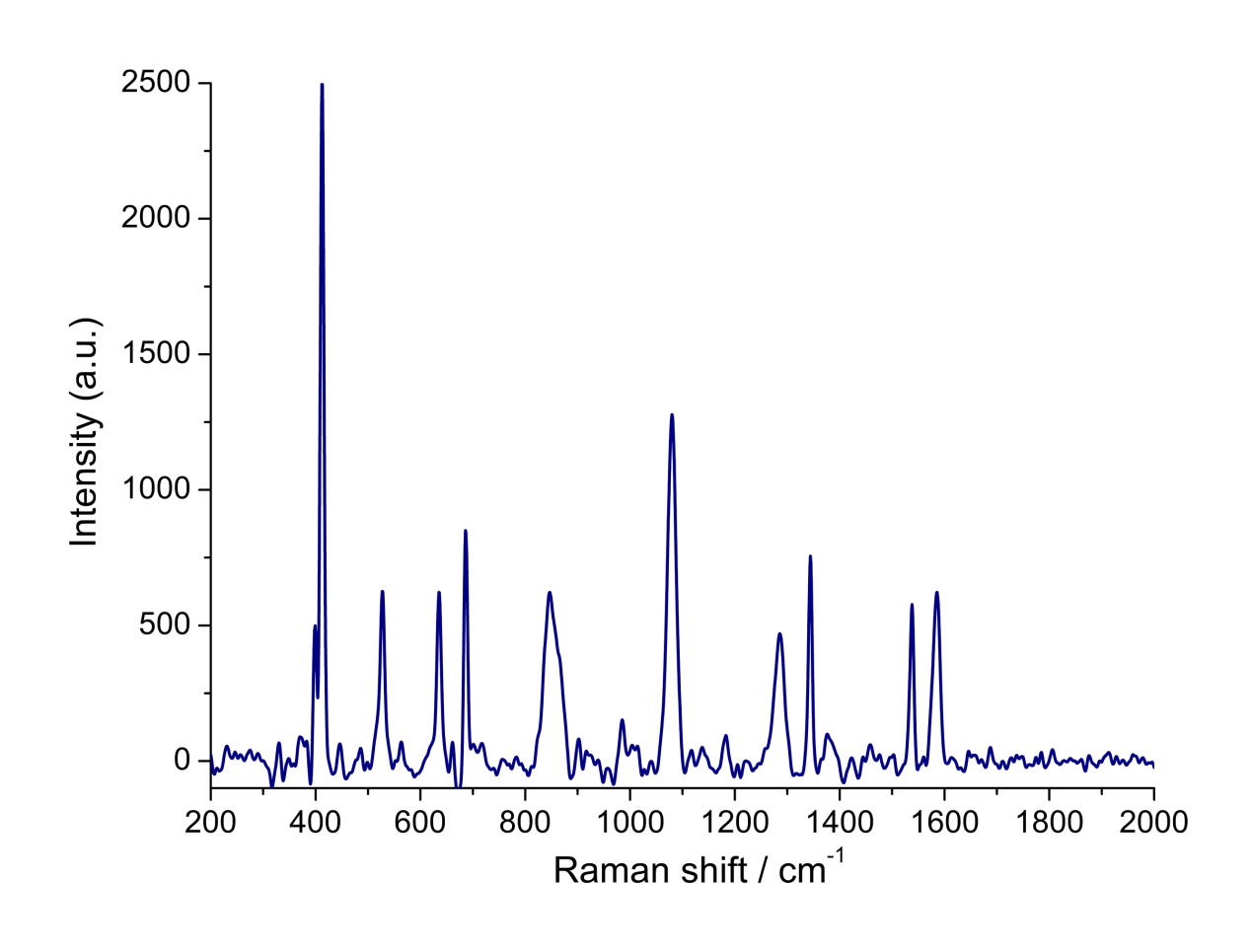


**Fig.S32** Raman spectroscopy of SERS ICSs detect 0.78 ng/mL Cd^2+^.


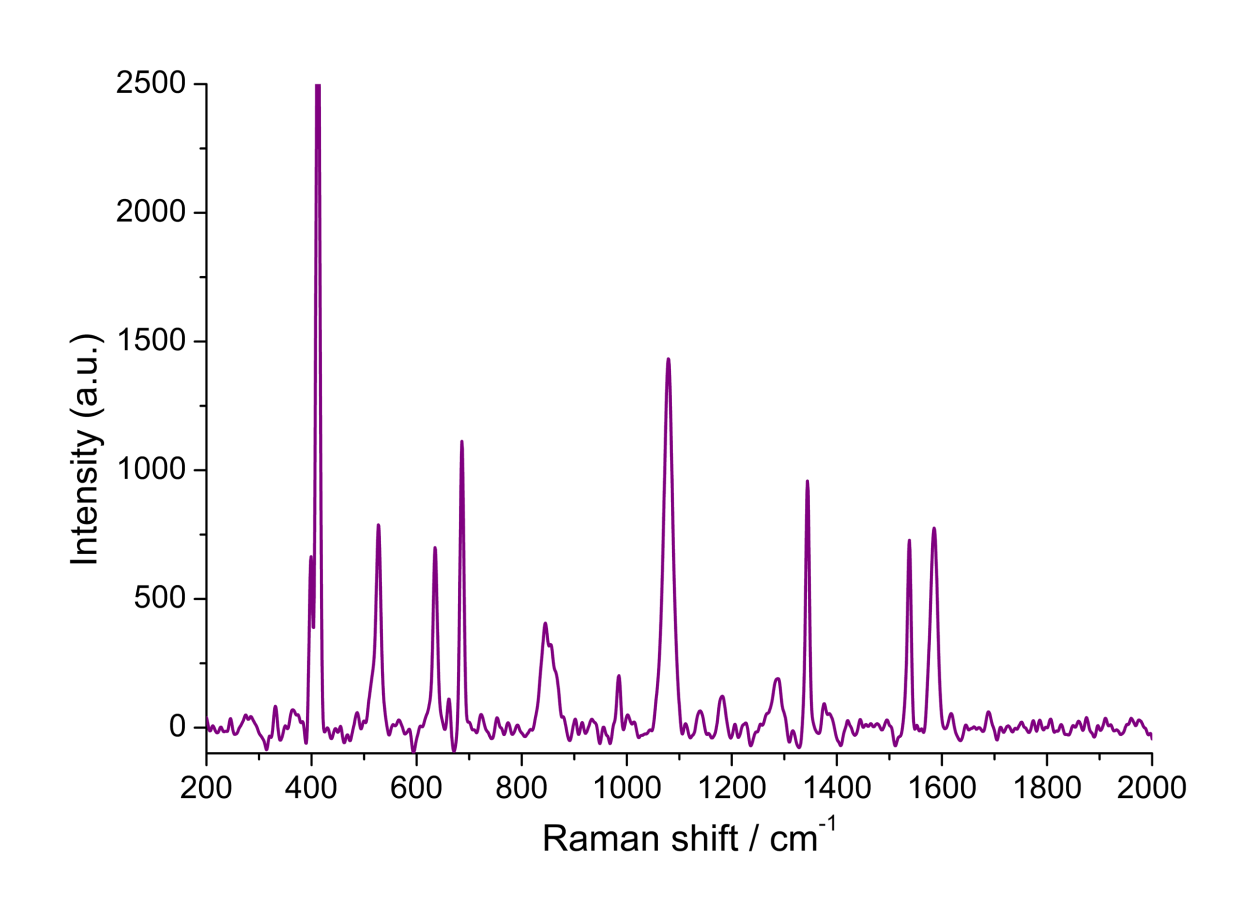


**Fig.S33** Raman spectroscopy of SERS ICSs detect 0.36 ng/mL Cd^2+^.


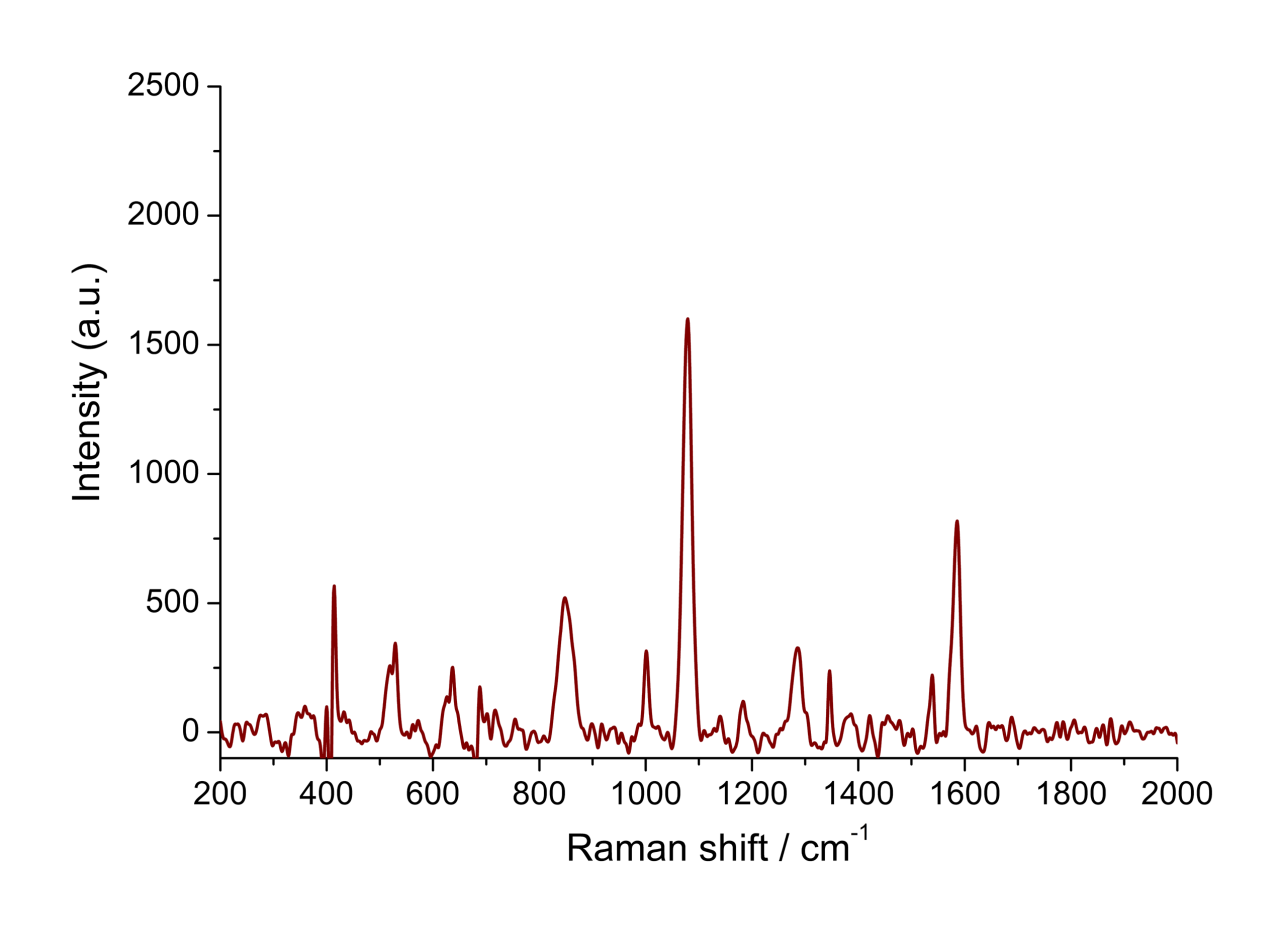


**Fig.S34** Raman spectroscopy of SERS ICSs detect 0.19 ng/mL Cd^2+^.


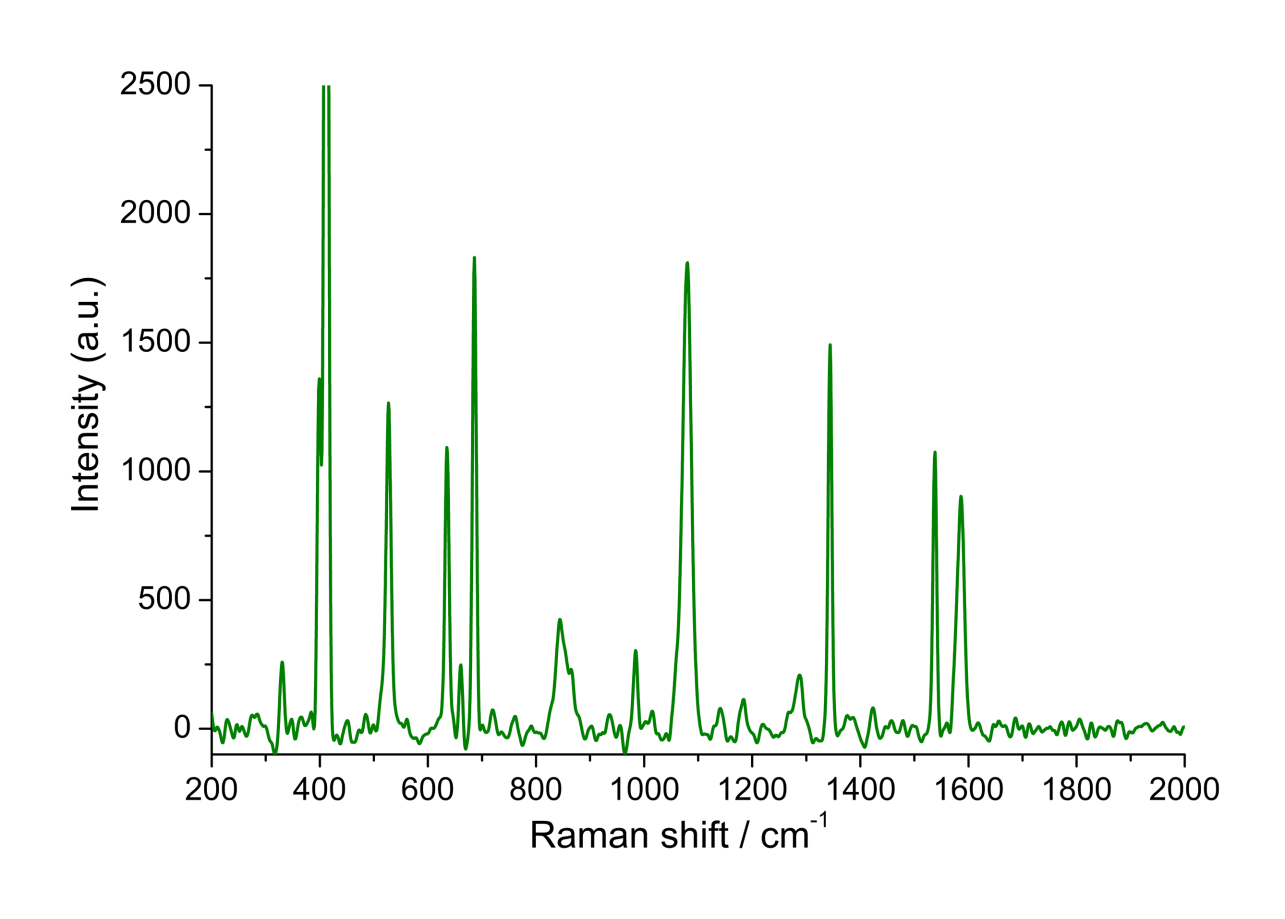


**Fig.S35** Raman spectroscopy of SERS ICSs detect 0.1 ng/mL Cd^2+^.


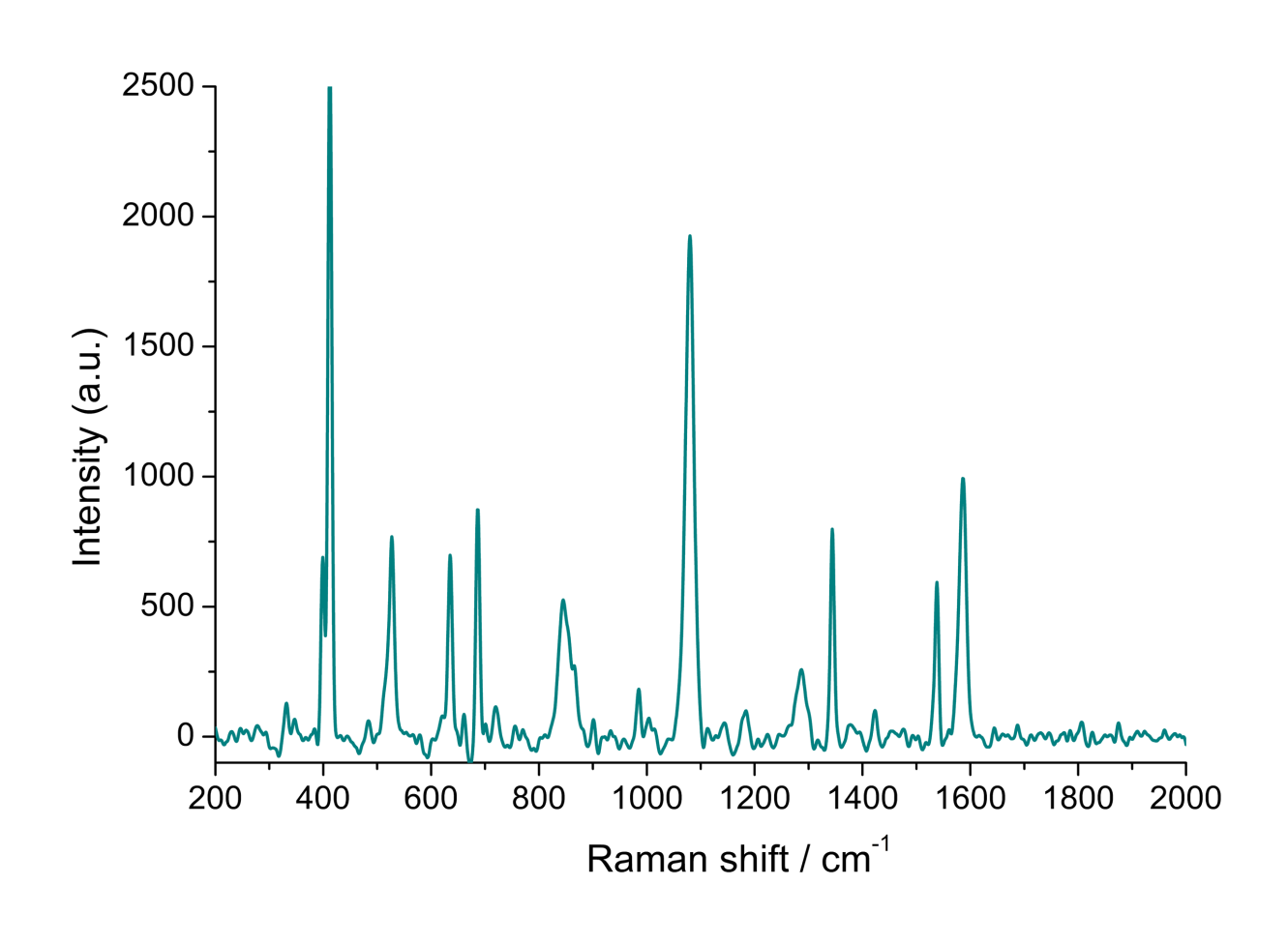


**Fig.S36** Raman spectroscopy of SERS ICSs detect 0.05 ng/mL Cd^2+^.


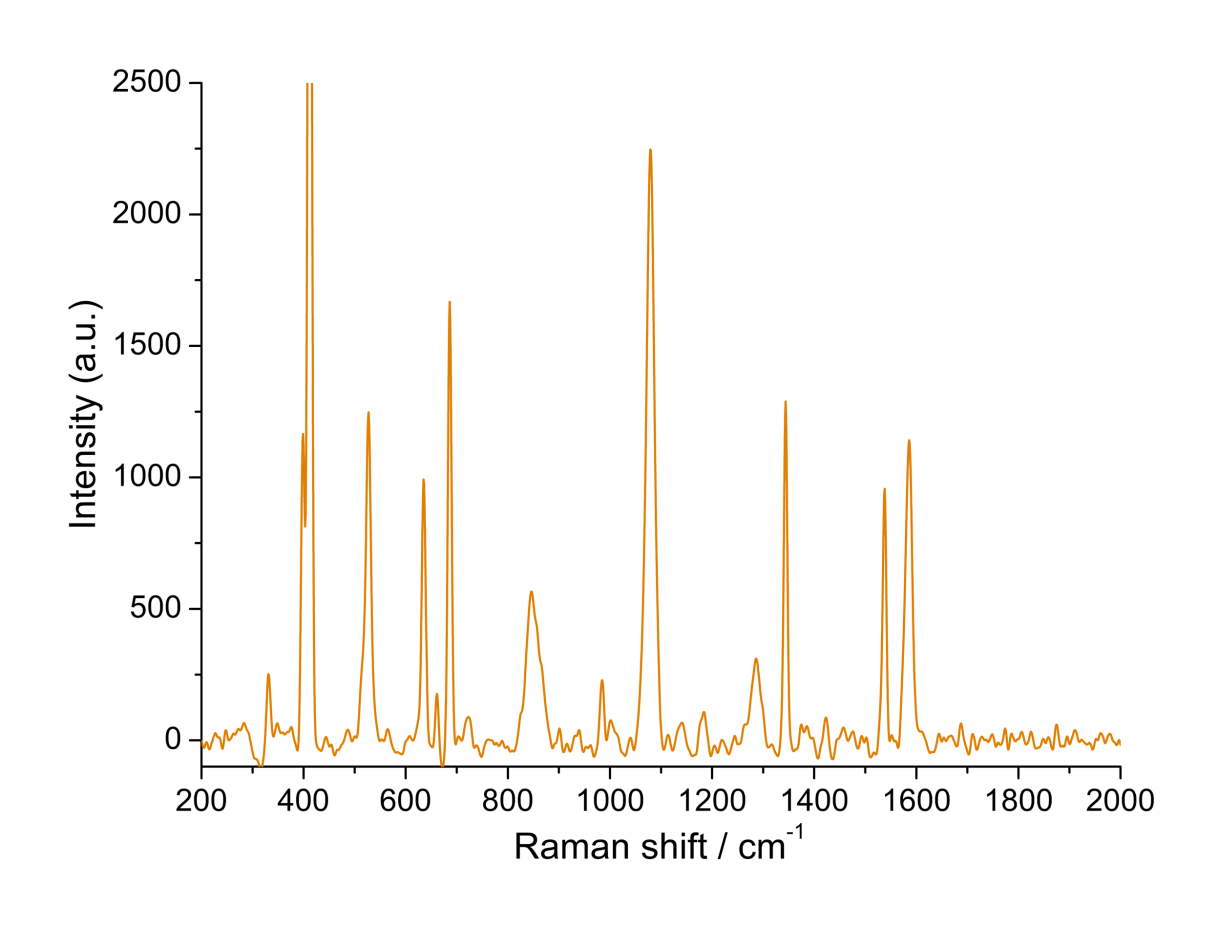


**Fig.S37** Raman spectroscopy of SERS ICSs detect 0.025 ng/mL Cd^2+^.


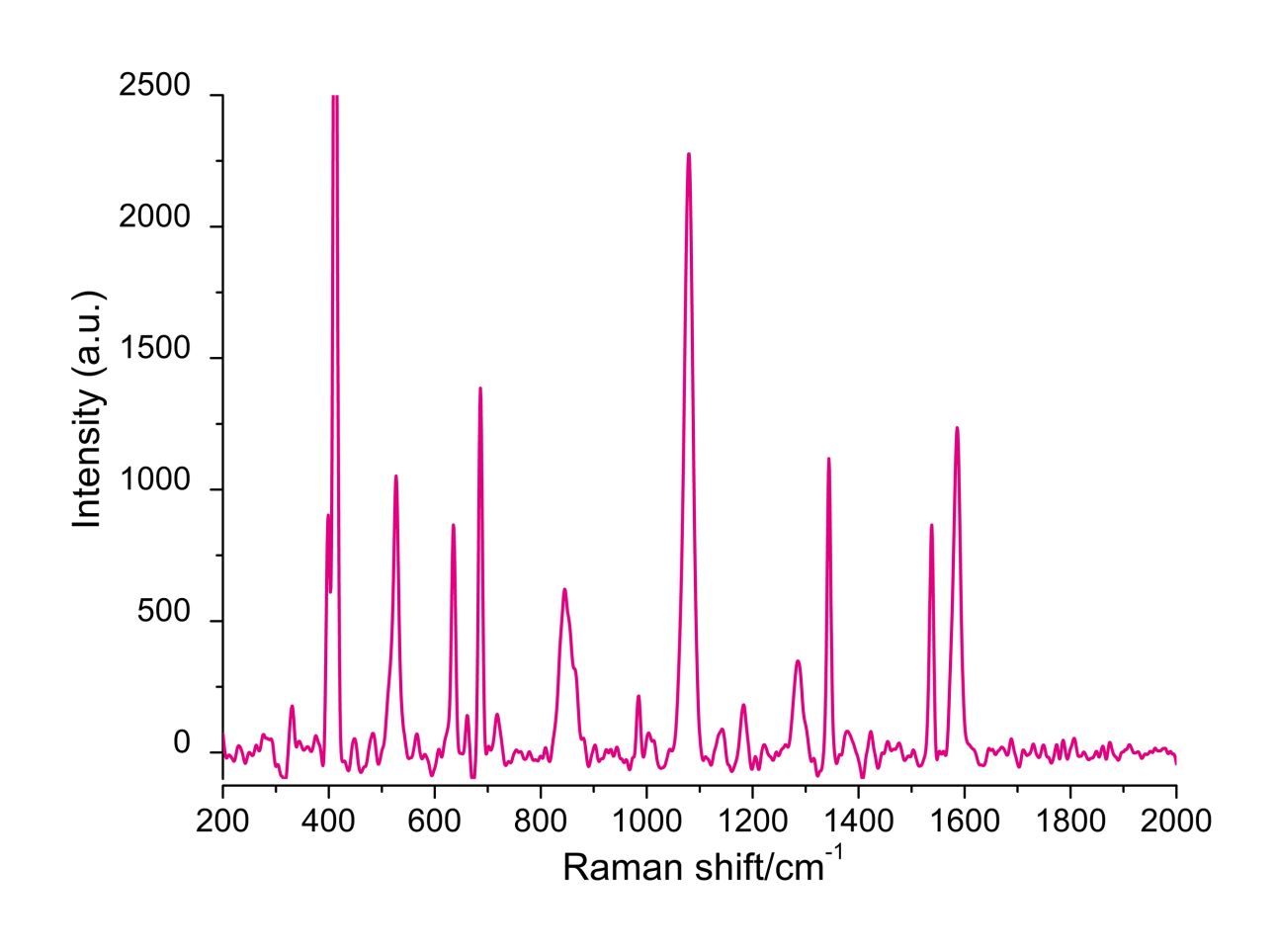


**Fig. S38** Raman spectroscopy of SERS ICSs detect 0 ng/mL Cd^2+^.

**Table S2.** Detailed Raman peaks of SERS ICSs for detect different concentration of Cd^2+^

| Concentration on Cd^2+^ (ng/ml) | 0 | 0.03 | 0.05 | 0.1 | 0.2 | 0.38 | 0.78 | 1.56 | 3.13 | 6.25 | 12.5 | 25 | 50 | 100 |
| --- | --- | --- | --- | --- | --- | --- | --- | --- | --- | --- | --- | --- | --- | --- |
| Raman peaks (1077 cm^-1^) | 2145 | 2135 | 2016 | 1797 | 1610 | 1488 | 1326 | 1045 | 766 | 425 | 249 | 95 | 91 | 95 |
| S.D. | 43.3 | 31.4 | 174 | 88.6 | 181.15 | 82.7 | 86.3 | 82.8 | 94.7 | 27.3 | 16 | 11.9 | 16.1 | 11.1 |
| Coefficient of variation (%)^c^ | 2.02 | uu | 8.64 | 4.82 | 11.50 | 5.56 | 6.51 | 7.93 | 12.37 | 6.44 | 6.44 | 12.56 | 17.71 | 11.75 |


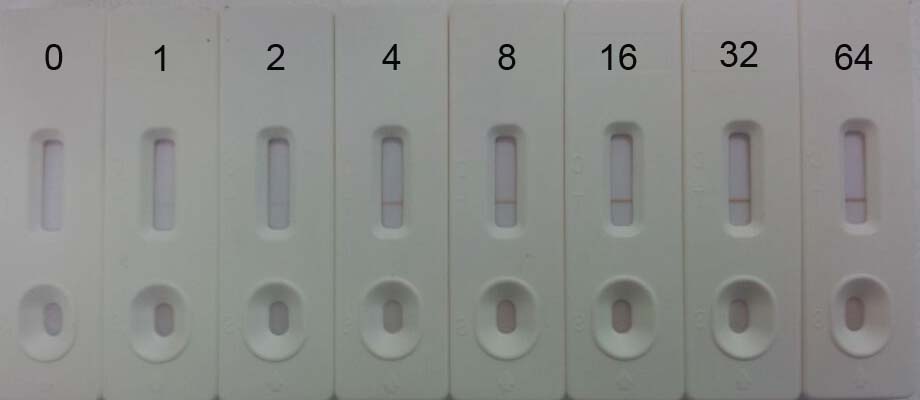


**Fig. S39. Result of AuNPs ICSs for detection of hemoglobin.**


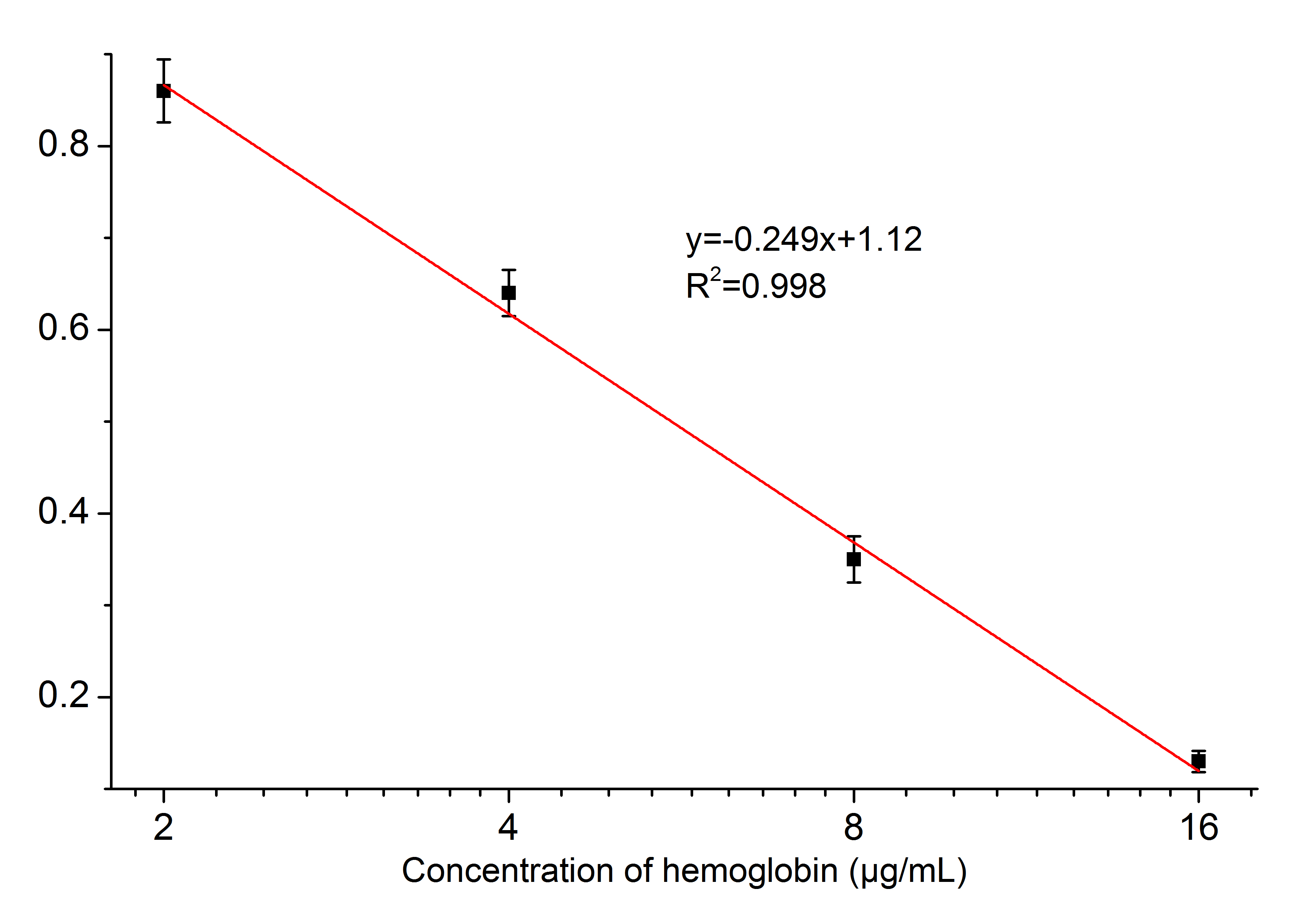


**Fig. S40.** The calibration curve of the AuNPs ICSs for detection of Cd^2+^. Each value presents the mean from 3 replicates (n = 3).


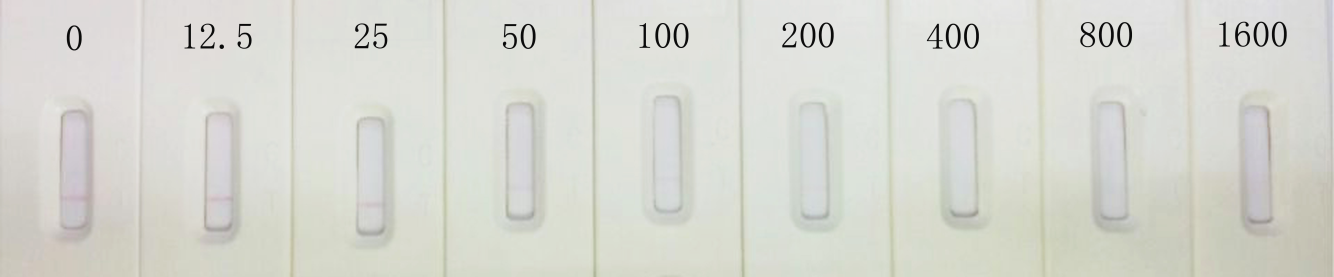


**Fig. S41. Result of AuNPs ICSs for detection of Cd^2+^.**


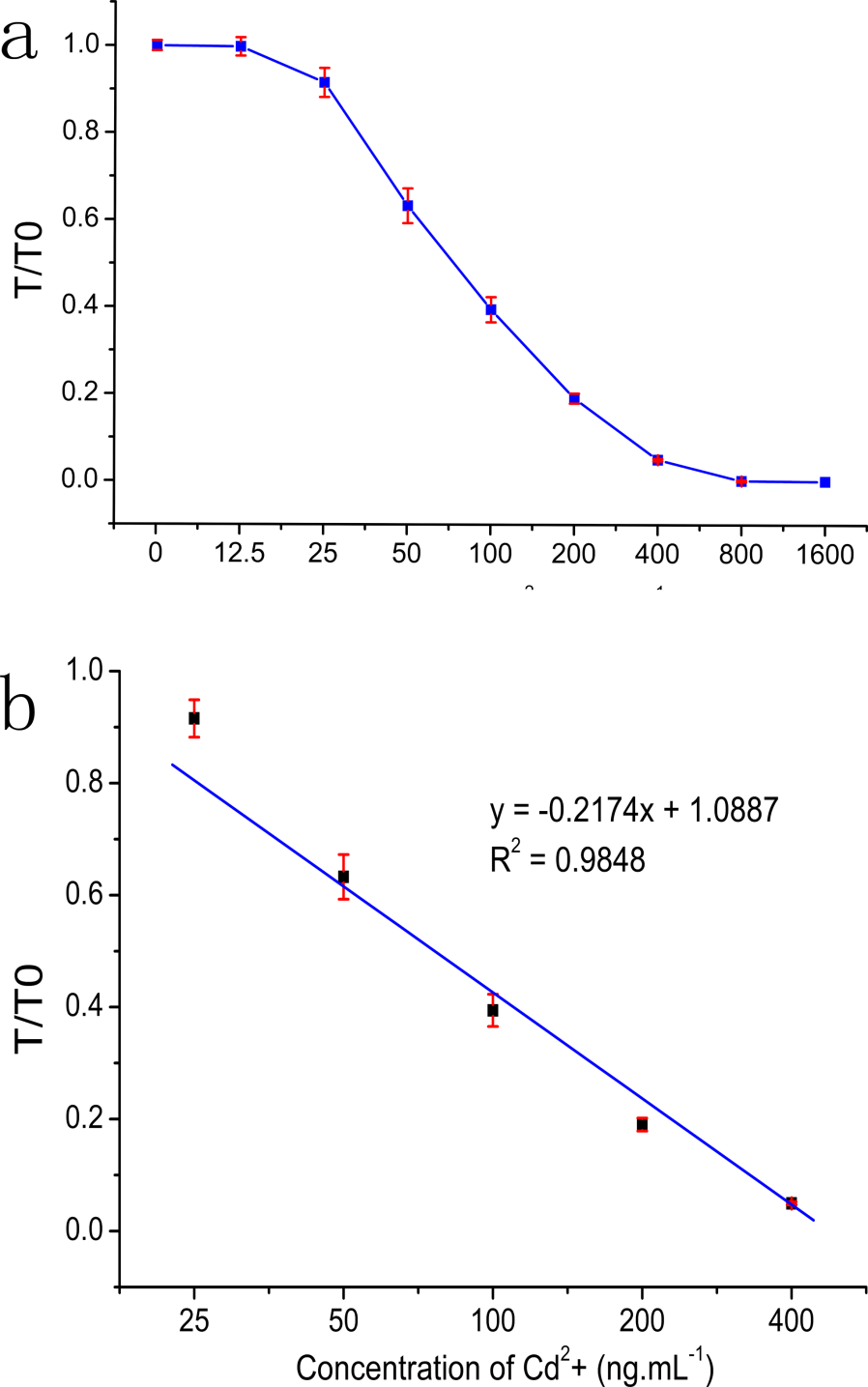


**Fig. S42.** The calibration curve of the AuNPs ICSs for detection of Cd^2+^. Each value presents the mean from 3 replicates (n = 3).


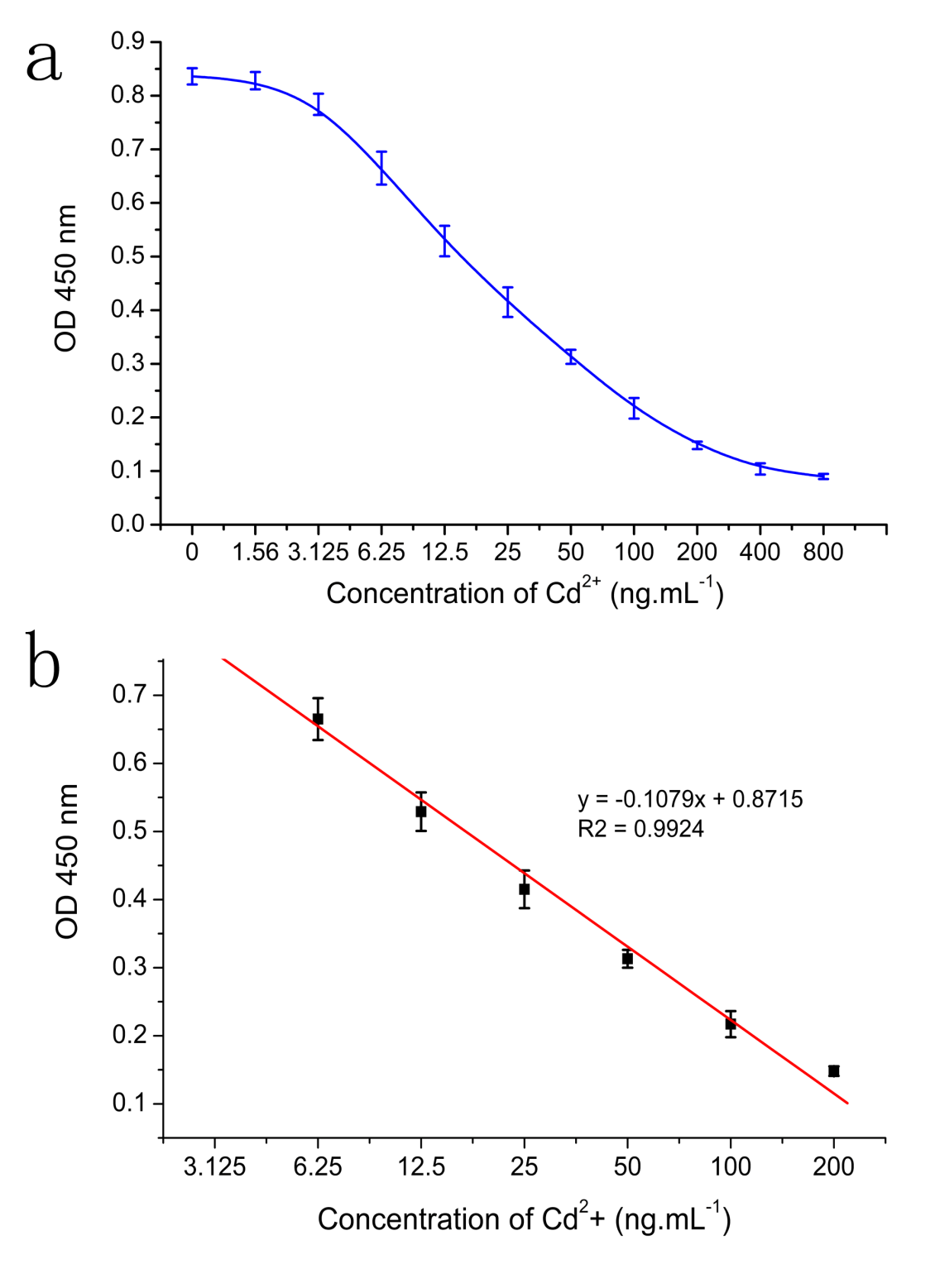


**Fig. S43.** The calibration curve of the ELISA for detect Cd^2+^. Each value presents the mean from 3 replicates (n = 3).

**Table3.** Current methods for detect haemoglobin

| Methods | Real sample | Limit of Detection | Reference |
| --- | --- | --- | --- |
| SERS immunochromatographic sensors | blood | 32 ng/mL | This work |
| photoluminescent gold nanodots sensor | blood | 1.0 to 10 nM | [[1](#_ENREF_1)] |
| high-performance liquid chromatography |  | 16.3 mg/mL | [[2](#_ENREF_2)] |
| electrochemical sensor | blood | 0.7 pM | [[3](#_ENREF_3)] |
| liquid-modified graphene based molecular imprinting electrochemical sensor | blood | 30.9 ng/mL | [[4](#_ENREF_4)] |
| Molecularly Imprinted Polymer Thin Film Based Surface Plasmon Resonance Sensor |  | 43.5 ng/mL | [[5](#_ENREF_5)] |
| hybrid organic–inorganic sensor | urine | 0.1 mg/ml | [[6](#_ENREF_6)] |

**Table S4.** Current methods for detect Cd^2+^ ion.

| Methods | Real sample | Limit of Detection | Reference |
| --- | --- | --- | --- |
| SERS immunochromatographic sensors | Environmental water and tap water | 0.05 ng/mL | This work |
| AuNPs immunochromatography | Grain | 0.2 ng/mL | [[7](#_ENREF_7)] |
|  | Environmental water and tap water | 10 ng/mL | [[8](#_ENREF_8)] |
| ELISA | Tap water and environmental water | 0.31 ng/mL | [[9](#_ENREF_9)] |
|  | Serum Sample | 0.24 ng/mL | [[10](#_ENREF_10)] |
| Microcantilever sensors |  | 0.11 ng/mL | [[11](#_ENREF_11)] |
| IC-ELISA | Electroplating Waste Water | 1.95 ng/L | [[12](#_ENREF_12)] |
| Fluorescence polarization immunoassay |  | 1.0 nM | [[13](#_ENREF_13)] |
| Aptamer sensor |  | 4.6 nM | [[14](#_ENREF_14)] |
| UV spectrophotometry | Water sample | 0.01 ng/mL | [[15](#_ENREF_15)] |
| Atomic absorption spectrometry (AAS) | bovine liver | 14 ng/L | [[16](#_ENREF_16)] |
| ICP-MS |  |  | [[17](#_ENREF_17)] |


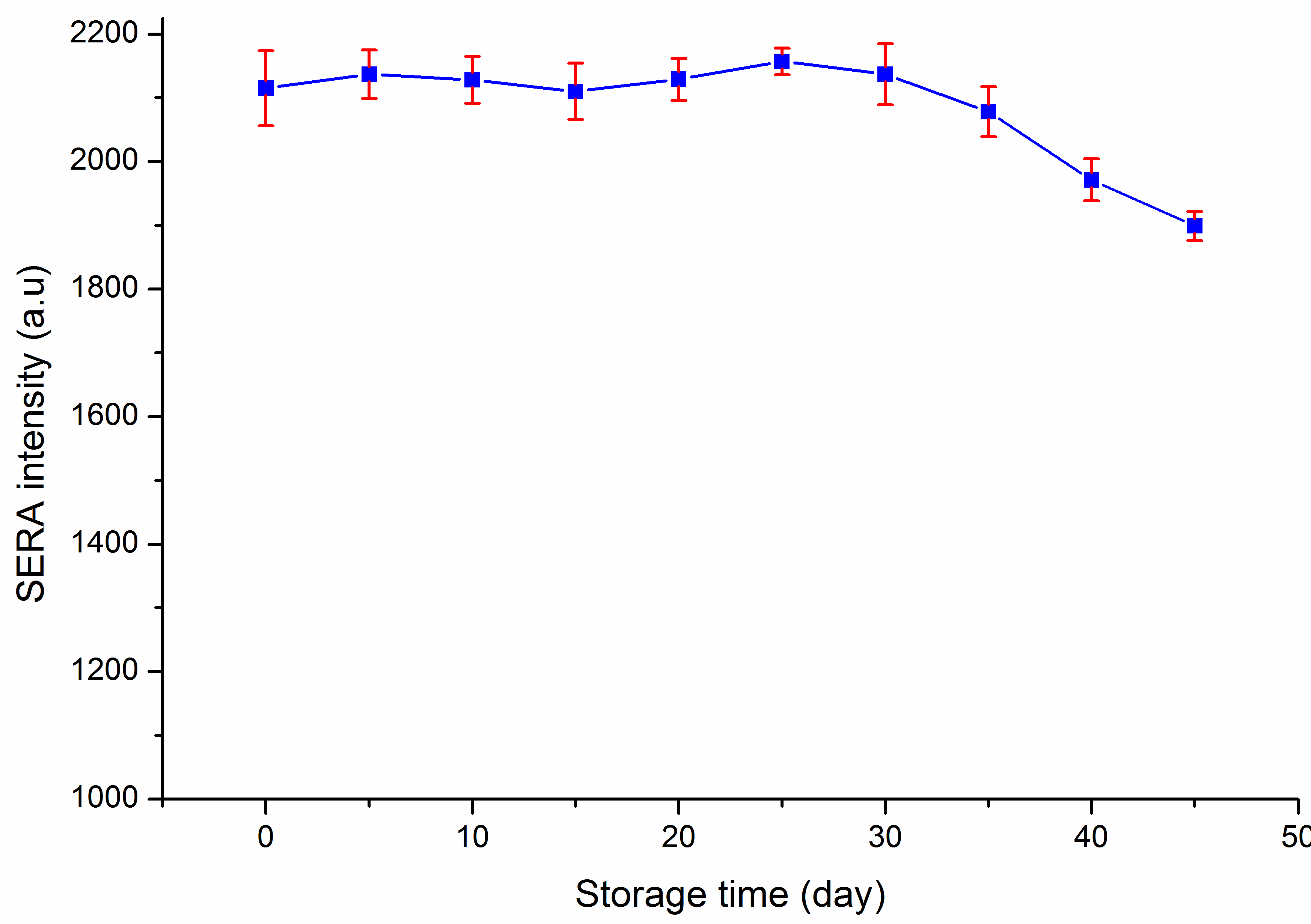


**Fig. S43.** Thermal accelerated test to study the storage time of SERS ICSs for detect Cd^2+^ ion. SERS ICSs was stored in 37 ℃ and SERS intensity of the ICSs keep constant 30 day. According to Arrhenius equation, the developed SERS ICSs could stored 120 days a t 25 ℃.

1. Chen LY, Huang CC, Chen WY, Lin HJ, Chang HT: **Using photoluminescent gold nanodots to detect hemoglobin in diluted blood samples.** *Biosensors & Bioelectronics* 2013, **43:**38-44.

2. Uzquiano M, Risin SA: **Premature neonate with apnea and trace amounts of hemoglobin S and A detected by high-performance liquid chromatography.** *Archives of Pathology & Laboratory Medicine* 2006, **130:**733-734.

3. Matysiak E, Donten M, Kowalczyk A, Bystrzejewski M, Grudzinski IP, Nowicka AM: **A novel type of electrochemical sensor based on ferromagnetic carbon-encapsulated iron nanoparticles for direct determination of hemoglobin in blood samples.** *Biosensors & Bioelectronics* 2015, **64:**554-559.

4. Wang ZH, Li F, Xia JF, Xia L, Zhang FF, Bi S, Shi GY, Xia YZ, Liu JQ, Li YH, Xia LH: **An ionic liquid-modified graphene based molecular imprinting electrochemical sensor for sensitive detection of bovine hemoglobin.** *Biosensors & Bioelectronics* 2014, **61:**391-396.

5. Wang Y, Zhang QW, Ren YM, Jing LJ, Wet TX: **Molecularly Imprinted Polymer Thin Film Based Surface Plasmon Resonance Sensor to Detect Hemoglobin.** *Chemical Research in Chinese Universities* 2014, **30:**42-48.

6. Tatikonda AK, Tkachev M, Naaman R: **A highly sensitive hybrid organic-inorganic sensor for continuous monitoring of hemoglobin.** *Biosensors & Bioelectronics* 2013, **45:**201-205.

7. Abe K, Nakamura K, Arao T, Sakurai Y, Nakano A, Suginuma C, Tawarada K, Sasaki K: **Immunochromatography for the rapid determination of cadmium concentrations in wheat grain and eggplant.** *Journal of the Science of Food and Agriculture* 2011, **91:**1392-1397.

8. Sasaki K, Tawarada K, Okuyama A, Kayama F, Abe K, Okuhata H, Maruyama Y, Arakane T, Miyasaka H, Fujikawa T, Ohmura N: **Rapid determination of cadmium in rice by immunochromatography using anti-(Cd-EDTA) antibody labeled with gold particle.** *Bunseki Kagaku* 2007, **56:**29-36.

9. Zhu X, Xu L, Lou Y, Yu H, Li X, Blake DA, Liu F: **Preparation of specific monoclonal antibodies (MAbs) against heavy metals: MAbs that recognize chelated cadmium ions.** *Journal of Agricultural and Food Chemistry* 2007, **55:**7648-7653.

10. Darwish IA, Blake DA: **Development and validation of a one-step immunoassay for determination of cadmium in human serum.** *Analytical Chemistry* 2002, **74:**52-58.

11. Velanki S, Kelly S, Thundat T, Blake DA, Ji H-F: **Detection of Cd(II) using antibody-modified microcantilever sensors.** *Ultramicroscopy* 2007, **107:**1123-1128.

12. Liu G-L, Wang J-F, Li Z-Y, Liang S-Z, Wang X-N: **Immunoassay for Cadmium Detection and Quantification.** *Biomedical and Environmental Sciences* 2009, **22:**188-193.

13. Johnson DK: **A fluorescence polarization immunoassay for cadmium(II).** *Analytica Chimica Acta* 1999, **399:**161-172.

14. Wu Y, Zhan S, Wang L, Zhou P: **Selection of a DNA aptamer for cadmium detection based on cationic polymer mediated aggregation of gold nanoparticles.** *Analyst* 2014, **139:**1550-1561.

15. Wen X, Yang Q, Yan Z, Deng Q: **Determination of cadmium and copper in water and food samples by dispersive liquid-liquid microextraction combined with UV-vis spectrophotometry.** *Microchemical Journal* 2011, **97:**249-254.

16. Junior MMS, Silva LOB, Leao DJ, Ferreira SLC: **Analytical strategies for determination of cadmium in Brazilian vinegar samples using ET AAS.** *Food Chemistry* 2014, **160:**209-213.

17. Pallavicini N, Engstrom E, Baxter DC, Ohlander B, Ingri J, Rodushkin I: **Cadmium isotope ratio measurements in environmental matrices by MC-ICP-MS.** *Journal of Analytical Atomic Spectrometry* 2014, **29:**1570-1584.
